# Accurate lattice parameters from 2D-periodic images for subsequent Bravais lattice type assignments

**DOI:** 10.1186/s40679-018-0051-z

**Published:** 2018-03-28

**Authors:** P. Moeck, P. DeStefano

**Affiliations:** Nano-Crystallography Group, Department of Physics, Portland State University, P.O. Box 751, Portland, OR 97201 USA

**Keywords:** Lattice parameter extraction, 2D-Bravais lattice type, Pseudo-symmetry, Metric specialization

## Abstract

Three different algorithms, as implemented in three different computer programs, were put to the task of extracting direct space lattice parameters from four sets of synthetic images that were per design more or less periodic in two dimensions (2D). One of the test images in each set was per design free of noise and, therefore, genuinely 2D periodic so that it adhered perfectly to the constraints of a Bravais lattice type, Laue class, and plane symmetry group. Gaussian noise with a mean of zero and standard deviations of 10 and 50% of the maximal pixel intensity was added to the individual pixels of the noise-free images individually to create two more images and thereby complete the sets. The added noise broke the strict translation and site/point symmetries of the noise-free images of the four test sets so that all symmetries that existed per design turned into pseudo-symmetries of the second kind. Moreover, motif and translation-based pseudo-symmetries of the first kind, a.k.a. genuine pseudo-symmetries, and a metric specialization were present per design in the majority of the noise-free test images already. With the extraction of the lattice parameters from the images of the synthetic test sets, we assessed the robustness of the algorithms’ performances in the presence of both Gaussian noise and pre-designed pseudo-symmetries. By applying three different computer programs to the same image sets, we also tested the reliability of the programs with respect to subsequent geometric inferences such as Bravais lattice type assignments. Partly due to per design existing pseudo-symmetries of the first kind, the lattice parameters that the utilized computer programs extracted in their default settings disagreed for some of the test images even in the absence of noise, i.e., in the absence of pseudo-symmetries of the second kind, for any reasonable error estimates. For the noisy images, the disagreement of the lattice parameter extraction results from the algorithms was typically more pronounced. Non-default settings and re-interpretations/re-calculations on the basis of program outputs allowed for a reduction (but not a complete elimination) of the differences in the geometric feature extraction results of the three tested algorithms. Our lattice parameter extraction results are, thus, an illustration of Kenichi Kanatani’s dictum that no extraction algorithm for geometric features from images leads to *definitive* results because they are all aiming at an intrinsically impossible task in all real-world applications (Kanatani in Syst Comput Jpn 35:1–9, 2004). Since 2D-Bravais lattice type assignments are the natural end result of lattice parameter extractions from more or less 2D-periodic images, there is also a section in this paper that describes the intertwined metric relations/holohedral plane and point group symmetry hierarchy of the five translation symmetry types of the Euclidean plane. Because there is no definitive lattice parameter extraction algorithm, the outputs of computer programs that implemented such algorithms are also not definitive. Definitive assignments of higher symmetric Bravais lattice types to real-world images should, therefore, not be made on the basis of the numerical values of extracted lattice parameters and their error bars. Such assignments require (at the current state of affairs) arbitrarily set thresholds and are, therefore, always *subjective* so that they cannot claim objective definitiveness. This is the essence of Kenichi Kanatani’s comments on the vast majority of computerized attempts to extract symmetries and other hierarchical geometric features from noisy images (Kanatani in IEEE Trans Pattern Anal Mach Intell 19:246–247, 1997). All there should be instead for noisy and/or genuinely pseudo-symmetric images are rankings of the relative likelihoods of classifications into higher symmetric Bravais lattice types, Laue classes, and plane symmetry groups.

## Introduction and background

Direct space imaging techniques such as scanning tunneling microscopy (STM) and (scanning (S) electron probe and high-resolution (HR) parallel illumination) transmission electron microscopy (TEM) provide nowadays atomic resolution in detected images on a routine basis [1–4]. STEM and HRTEM images are typically projections from the third dimension and more or less 2D periodic when crystals are involved. Statistical precision of down to a few picometers is obtained in the case of STEM imaging [1]. This allows for “parametric model based imaging” [2, 3] where the accuracies and precisions of extracted structural-geometric image parameters are statistically estimated on the basis of information theory (i.e., maximum likelihood, negative Boltzmann entropy [5] or maximum log-likelihood [6] methods) and geometric inferences [7] are possible.

The information theory approach to the analysis of more or less 2D-periodic images is quantitative and considers microscopes as channels through which human beings obtain structural information about solids at the atomic level. The images that the microscopes deliver are “data planes” [2, 3] from which quantitative structural-geometric information is to be extracted (rather than to be interpreted visually in a more qualitative way). Local materials structure–property relationships can be extracted with this kind of approach [4] from scanning probe microscope (SPM) images that are atomically resolved. Extracted structural-geometric information is to be combined with what is obtainable from associated spectroscopic techniques and density functional theory [8] calculations in order to facilitate progress towards the developing knowledge-based “design of new materials” paradigm [9].

With some loss of statistical precision, 3D-atomic coordinates and elemental identities can also be determined nowadays by STEM from highly defective (poly-phase and poly-orientation) nanocrystals by means of “atomic electron tomography” utilizing for example 68 different 2D projections [10]. Small individual organic molecules such as oleic acid, CH_3_(CH_2_)_7_CH=CH(CH_2_)_7_COOH, could possibly be imaged in the future with a low electron dose in 3D-atomic resolution by electron exit wave reconstructions from HRTEM through-focus series (i.e., in-line holography) for which the individual images were recorded with parallel illumination either in a single projection [11] or, at most, in a few projections.

The information in the recorded data planes [2, 3] is often what is to be modeled (rather than details of the imaging process) so that extraction algorithms become largely independent of the type of microscope with which the data has been recorded [4, 12, 13], see also footnote.^1^ As a matter of fact, one may view much of the astonishing progress in atomic resolution STEM and HRTEM of the last few decades as taking the information scrambling effects of the microscope hardware to a large extent out of recorded data. Note in passing that the associated reduction of model parameter space dimension developments [14] along with the emergence of the quantitative evidence/knowledge-based materials design paradigm and the treatment of images as data planes [15] were all foreseen some two decades ago.

Unavoidable noise in the imaging process of more or less 2D-periodic arrays of physical objects is a problem because it obscures the signal and limits the statistical accuracy and precision of extracted structural-geometric parameters [1–4, 12]. When systematic imaging errors are negligibly small in comparison to random errors and the amount of approximately Gaussian noise due to the imaging process is also reasonably small, one is justified in utilizing geometric Akaike information criteria (G-AICs) [7, 16–18] for the ranking of evidence in favor of scientific hypotheses with respect to their relative likelihoods. Both the “accuracy/disagreement” and the “generality/sophistication” of the models that represent these hypotheses are taken into account in an appropriate manner by these criteria. A corollary of this approach is that no geometric feature extraction algorithm will ever deliver *definitive* results in real-world applications [7, 16].

With real-world applications we mean all kinds of applications where noisy experimental data of finite resolution is involved, rather than abstract geometric entities. One is, however, typically able to identify the geometric-structural model that represents the desired aspect of image data with a minimum of information loss [5, 6]. Relative likelihood ratios [6, 18], which represent the strength of quantified evidence in favor of one model (or hypothesis) with respect to another, can always be calculated on the basis of traditional [6] and geometric AICs [7]. So-called “Akaike weights” [6, 18] represent the probability that a geometric-structural model minimizes the unavoidable information loss when it is selected to represent experimental data. These weights are also useful for multi-model inferences and predictions. They can also be summed up into confidence sets. Individual Akaike weights and their confidence sets allow for noise-level dependent quantitative spreadings of crystallographic symmetry classifications over several classes [18] in databases. In the case of crystallographic symmetry classifications, one can combine Akaike weights for classifications into Bravais lattice types, Laue classes, and plane symmetry groups in order to make the total classification comprehensive [18].

As there are *no definitive* geometric-structural feature extraction algorithms for noisy images, the results of different computer programs that represent these algorithms are to be compared to each other in order to gain insights into their robustness with respect to the presence of noise and also their reliability with respect to subsequent geometric inferences such as the assignments of Bravais lattice types. The main thrust of this paper is, however, not the comparison of the relative performance of three different algorithms/computer programs [19–21] with respect to the task of 2D-lattice parameter extractions from four sets of synthetic test images [19]. Our main thrust is instead to utilize the performance comparisons of these geometric feature extraction algorithms as illustrations of Kenichi Kanatani’s dictum that all extraction algorithms for geometric-structural features from images are aiming at an intrinsically impossible task in all real-world applications.

The assignment of Bravais lattice types, i.e., qualitative classifications, are the natural end result of quantitative lattice parameter extractions from more or less 2D-periodic images. In a follow-up paper that is to be published elsewhere, we will utilize a recently developed G-AIC [17, 18] for the classification of the extracted lattice parameter sets of this paper into Bravais lattice types. We will also provide the respective Akaike weights and confidence sets for different translation symmetry hierarchy branches there.

In the present review, we will only allude to the fact that the assignment of higher symmetric Bravais lattices to extracted lattice parameter sets on the basis of their error bars (and by means of null hypothesis tests) is not optimal because the results are bound to be in error insofar as they claim to be *definitive*. This is because of three reasons: (i) the intertwined holohedral [22] point/plane symmetry and metric relation hierarchy of the 2D-Bravais lattices types (that will be described in detail in the following section), (ii) the need for arbitrarily set thresholds in order to deal with symmetries that are unavoidably broken by noise, and (iii) possibly existing (genuine) pseudo-symmetries of the first kind [23] and metric specializations [24], see also footnotes^2^ and 3 for more explanations on the latter two concepts.

Note that the five Bravais lattice types of the Euclidean plane [25, 26] constitute an exhaustive set of translation symmetry models in 2D. All complete lattice parameter sets that were extracted from more or less 2D-periodic images can, therefore, always be classified with a maximized likelihood as corresponding to one of these Bravais lattice types. Any traditional distance measure between extracted lattice parameter sets and the five translation symmetry models that is not properly balanced by accounting for the number of fitting parameters will always be smallest for the least symmetric Bravais lattice. This is the essence of Kenichi Kanatani’s two decades old comments on the state of the art of automatic detections of symmetries in noisy images [27] and an unavoidable consequence of the hierarchy of the translation symmetries in the Euclidean plane.

**Fig. 1:**
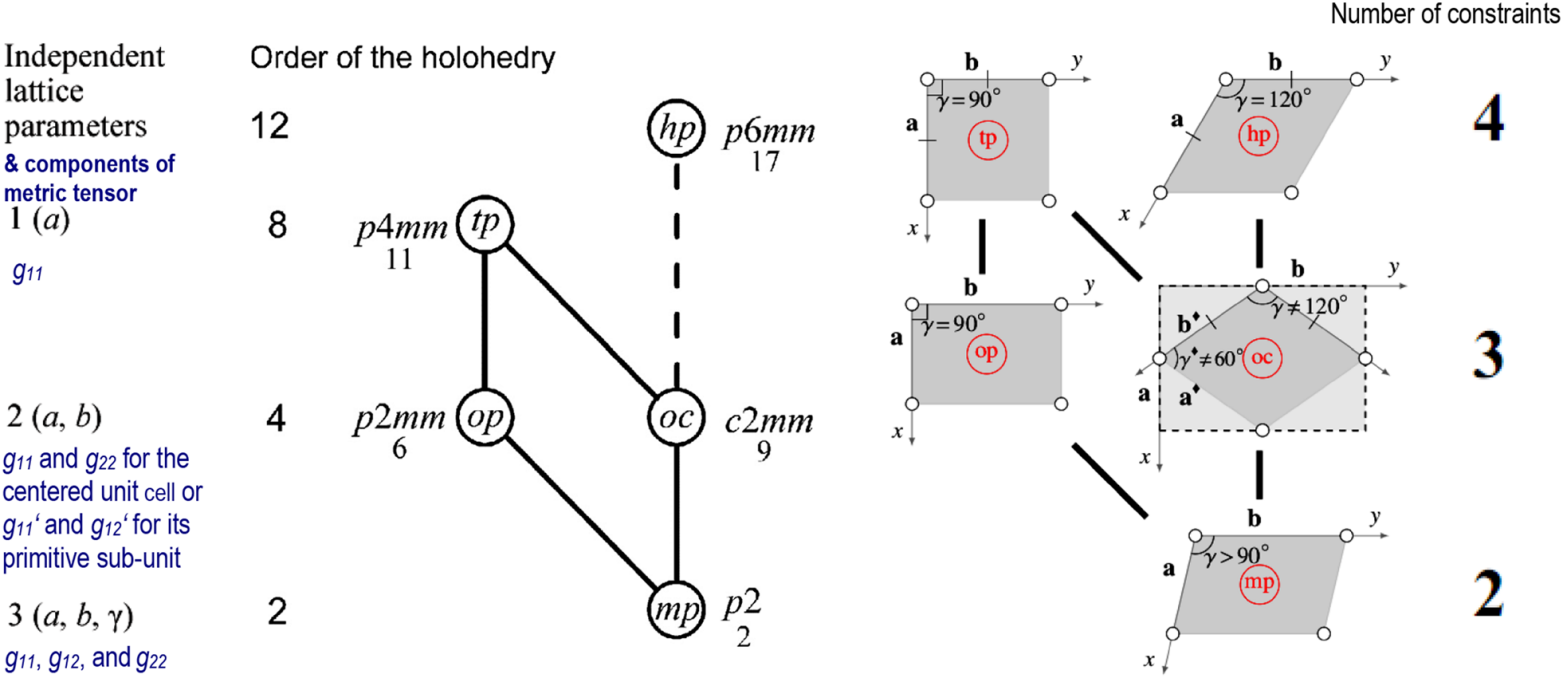
Aspects of the intertwined hierarchy of 2D-Bravais lattice types (modified after Refs. [17, 28, 29]). From the bottom to the top of this figure, the number of independent lattice parameters (most to the left, which is also the number of independent components of the metric tensors) decreases while the number of geometry/symmetry constraints (bold large font numbers most to the right) increases. The plane symmetry hierarchy of the Bravais lattice types is illustrated by the middle-left sketch. The type of Bravais lattice at the upper end of a line in this sketch is a special case (metric specialization) of the type at its lower end. Solid lines indicate ordinary subgroups in this sketch, the dashed line stands for a set of three conjugate plane symmetry subgroups. The plane symmetry groups of the Bravais lattice types (also known as the holohedries) are explicitly given by their symbol and number in Ref. [25], e.g., *p2* and number 2. The two letter symbols within the nodes of this sketch are the standard abbreviations of the 2D-Bravais lattice types, e.g., *mp*. The order of the plane symmetry groups of the Bravais lattice types is given to the left of this sketch and corresponds to the multiplicity of the general position within these groups [25]. The middle-right sketch shows the related hierarchy of the primitive unit and sub-unit cell shapes. Short lines that are perpendicular to the basis vectors mark congruence (equal length) in this sketch. The ^♦^ sign signifies the parameters of the primitive sub-unit of the conventional rectangular centered unit cell. All four primitive unit cells and the primitive sub-unit of the *oc* Bravais lattice possess the same area in this sketch. The number of geometric (metric and symmetry) constraints on the unit and sub-unit cells has been taken from Refs. [17, 18] and is further elaborated on in Table 1

The intertwined metric relation and holohedral point/plane symmetry-based hierarchy of the Bravais lattice types [28, 29] forms the backbone of both the underlying geometric model selection process and our recently developed G-AIC procedure [17, 18] that allows for subjective threshold-free Bravais lattice type classifications at the given noise level of a more or less 2D-periodic image. For some future 2D-periodic images with smaller noise levels, the relative likelihoods of assigned higher symmetric Bravais lattices may change somewhat. Smaller noise levels per unit cell can, for example, be obtained by the processing of larger image areas with significantly more repeats of the unit cell. We will expand on all of this elsewhere (in other papers) but feel compelled to discuss Bravais lattice types in 2D in the following “Bravais lattice types in two dimensions” section of this paper, see Fig. 1 and Table 1, because there are related misconceptions in the wider scientific community, e.g., [30–32], and even misrepresentations in the scientific literature [33].

A clearing up of these misconceptions is a secondary thrust of this paper and important in its own right to support further developments of algorithms for the extraction of geometric-structural parameters from more or less 2D-periodic images. This is because there is much more information on the underlying geometry/structure and symmetry in noisy STEM and HRTEM images of nearly ideal single crystals [34, 35] and bicrystals [36] to be extracted for “structural fingerprinting purposes” than just lattice parameters and Bravais lattice types.

Note that Refs. [4, 12], for example, constitute significant progress over the current state of the art as they fit into the “big, deep, and smart data” schemes of the developing materials design approach [9]. Reference [4] describes, however, only the extraction of lattice parameters as structural identifiers for the spatial location of crystals with different phases that are present in the same sample. An underlying assumption of the technique in Ref. [4] is linear imaging, which, while justified for STM and SPM, could be undermined by dynamical scattering effects in the case of electron microscopy. Nevertheless, a physical-structural model-based image feature extraction technique that was developed for one type of microscope has in Ref. [4] been transferred to another type of microscope. (The same kind of thing is stated in Ref. [12] and described in Ref. [13]).

In the supplemental material to their paper, the authors of Ref. [4] mention that classifying extracted lattice parameter sets into Bravais lattice types would be a useful extension to their algorithm. In their paper itself, these authors mention also the technical possibility of extracting local plane symmetry groups by using their sliding fast (discrete) Fourier transform windows approach (see also Refs. [12] and [37]), but caution that this *“would require substantial efforts at developing the appropriate image classification schemes”* [4].

These kinds of classification schemes should ideally be a combination of the translation and site^4^ symmetry parts of plane symmetries and based on G-AICs [7, 16–18] in order to avoid arbitrarily set thresholds. This is because threshold-free translation symmetry classifications can be based solely on the maximal likelihood position of a few Fourier coefficients (FCs) in the amplitude map of the discrete Fourier transform (dFT) of a more or less 2D-periodic image [17]. Threshold-free classifications of plane symmetries require, on the other hand, the knowledge of the intensity values of all pixels (in real space) [30] or of the amplitudes and phase angles of all FCs of such images (in reciprocal space) [18], but not the FC positions in the dFT amplitude map. It is the combination of these two kinds of information that leads to the plane symmetries that need to be classified.

While a certain set of site symmetries constitutes a point symmetry group in the Euclidian plane and requires a compatible translation symmetry type, a certain translation symmetry type enables a few sets of site symmetries in 2D-periodic images. For example, the site symmetries of plane symmetry groups *p4*, *p4mm* and *p4gm* all require a square lattice. The square Bravais lattice type, on the other hand, enables three sets of site symmetries when (structure-less) lattice points^5^ are expanded into 2D-periodic motifs, which are either symmorphic or non-symmorphic [25, 26]. These intertwined relationships between site/point and translation symmetries in the Euclidean plane are further alluded to in the following section and can be utilized to validate Bravais lattice type assignments on the basis of extracted lattice parameters by the independent route over the compatible plane symmetry groups.

Because point and translation symmetries are intertwined in the crystallographic description of crystals based on more or less periodic images that were taken of them, a Bravais lattice type has been correctly assigned (in a qualitative way) only when the metric lattice parameter relations of Table 1 are obeyed within error bars and the site symmetries of the 2D-periodic motif are also compatible with this assignment [25]. The obeying of the metric lattice parameter relations are thereby quantitative measures and the compatible plane symmetry groups are an additional qualitative requirement that needs to be obeyed. We will expand on this elsewhere.

Geometric AICs that utilize particularly simple geometric models exclusively, i.e., work in a model parameter space of a rather small number of dimensions, become employable when microscopes are so good that essentially the same image is obtained almost all of the time under the same nominal imaging conditions [7, 16–18]. In other words, systematic errors need to be so small that they can be safely neglected with respect to random errors that also need to be reasonably small because G-AICs are first order approximations.

The general route towards reaching the full potential of geometric-structural/physical model-based imaging of crystals in STEM, HRTEM, STM, and SPM might be a combination of the statistical approach outlined in papers such as Refs. [2, 3] with G-AICs [7, 16–18] and relative likelihood ratios/model probabilities [6]. Complementing aspects of the information in the recorded data are, thereby, to be modeled with complementing model sets such as compatible Bravais lattice types, Laue classes [38] (see also footnote^6^), and plane symmetry groups.

**Fig. 2:**
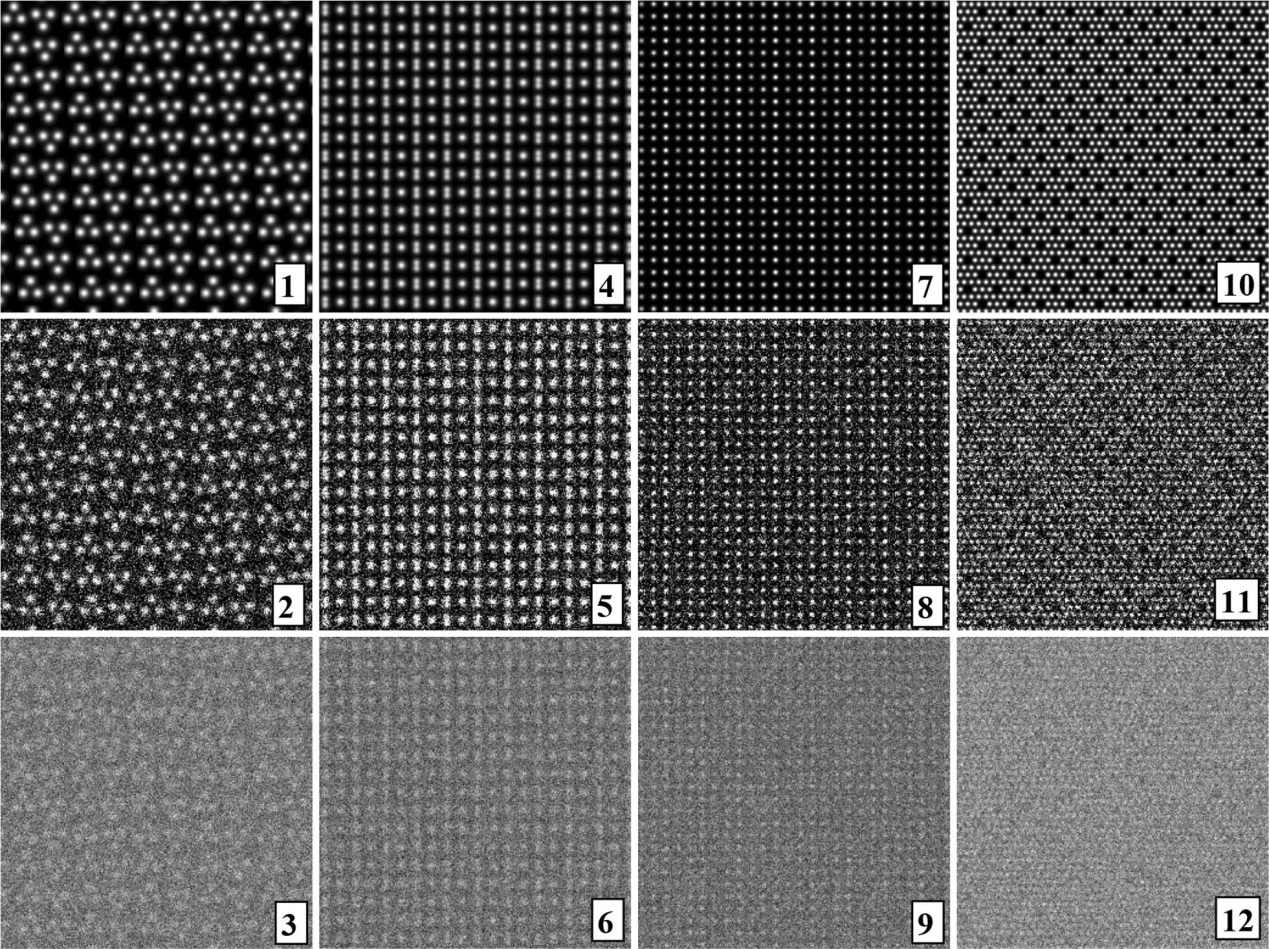
Sets of synthetic (256 times 256 pixel) test images arranged in columns where the first image is the one without noise and the second and third images were created by adding independent Gaussian noise of mean zero and a standard deviation of 10 and 50% of the maximal pixel intensity to the corresponding image in the top row. The images are numbered to provide for straightforward references below. Note that there are pronounced motif-based and translational pseudo-symmetries of the first kind in the test images #7 to #9. (In the two noisy images of this series, i.e., #8 and #9, as well as in all noisy images of the other three series, there are of course pseudo-symmetries of the second kind due to the addition of noise to the noise-free images in the top row.) Because image #10 features a metric specialization (see footnote 3), images #11 and #12 feature pronounced translational pseudo-symmetries of a “special” (see footnote 2) kind. Genuine pseudo-symmetries, the effects of added Gaussian noise on genuine symmetries, and the metric specialization lead to a somewhat “squarish” visual appearance of the images #7 to #12 and present challenges to lattice parameter extraction algorithms

In addition to the directly following more educational section on Bravais lattice types, Fig. 1 and Table 1, the rest of the paper comprises five more sections and is organized as follows. Information on the synthetic test images [19], Fig. 2, is collected in the “Overview I: synthetic test image sets” section of this paper. After that follows a discussion of the algorithms/computer programs [19–21], Table 2, which we employed in this review. This is followed by a brief section on particulars of our lattice parameter extraction procedures. The “Results and discussions” section presents the main results of this review, Tables 3, 4, 5 and 6, and their discussions. Finally we end this paper with a “Summary and conclusions” section.

## Bravais lattice types in two dimensions

A widespread misconception about 2D-Bravais lattice types in the wider scientific community is that they are considered to be independent of the origin and site symmetries of the plane symmetry groups. In Refs. [30, 31], for example, Bravais lattice types are considered to exist without any spatial relationships to the site symmetries of the motifs of the processed images that are more or less 2D periodic. (In Refs. [30, 32], the same has been done for 1D-periodic time series).

This practice ignores the origin conventions of the plane symmetry [25] (and subperiodic frieze [39]^7^) groups. As a matter of fact, the origins of higher symmetric plane symmetry groups (i.e., all groups higher than *p1* and *pg*) are indeed fixed by site symmetries of the motif higher than the identity operation. Depending on the plane symmetry group, the origin is either located at a point with a specific site symmetry (higher than the identity operation) or anywhere along a line where the site-translation symmetry combination is the same for each point on the line. Plane symmetry group *p1* is, thus, the only group without an origin convention [25]. The origin should never be arbitrarily chosen because that deprives one from utilizing the totality of the mathematical relationships between geometric-structural features in 2D (and 1D) periodic images, which are indispensable for comprehensive crystallographic classifications.

When the goal of a study is, however, not a comprehensive crystallographic classification as, for example, in numerous works in computational symmetry that are reviewed in Ref. [31] or the Primitive Unit Cell Extraction of Ref. [19], the plane symmetry group origin does not need to be specified. A recent computational symmetry study that aimed at comprehensive crystallographic classifications resulted in an appropriate origin choice^8^ as a byproduct [40]. Arbitrarily set thresholds needed to be utilized in order to assign mathematically exact symmetries to conspicuous “pseudo-symmetric features” in that study. This has, unfortunately, been so far the common practice in the computational symmetry as well as applied crystallographic image processing communities with very few notable exceptions, e.g., [17, 18, 30, 32].

Also confusing to the wider scientific community [33] is the existence of the rectangular centered Bravais lattice type in 2D while virtually every materials scientist or electron microscopists will be perfectly comfortable with the body-centered cubic Bravais lattice type and the tungsten structural prototype in 3D. In both cases, it is the underlying space symmetry of the lattice points (or W, Fe, Cr, Rb,…atoms) that allows for the centerings with the consequence that the conventional unit cells [25] contain two lattice points (or W atoms in case of tungsten), rather than one lattice point (or one W atom) that represents the primitive sub-unit cell. As this section of the paper is about clarifying crystallographic core concepts that are often misunderstood by the wider scientific community, it is fitting to recall key differences between lattice points and atoms or groups of atoms, in footnote 5.

Note that the primitive sub-units and the centered (i.e., conventional [25]) unit cells possess the same space symmetry. This symmetry just becomes more obvious in the case of the centered cells and, therefore, more useful as classification tool (albeit at the minor “intellectual expense” of larger unit cells). What is different between the two representations of the same space symmetry is that an alternative choice has been made for the translation symmetry part of the space group symmetry.

This is illustrated below by the discussion of the concepts of translational pseudo-symmetry [23] and metric specialization [24] for the example of the rectangular centered Bravais lattice type in 2D. We will use this example as well for the discussion of translation symmetries in reciprocal (Fourier) space.

There is an intertwined hierarchy of the 2D-Bravais lattice types based on both their metric properties and the holohedral plane symmetry groups. Figure 1 sums this hierarchy up. Table 1 gives an overview of the Bravais lattice types of the Euclidian plane and provides information on the metric property part of this hierarchy [17, 28, 29].

It is a common misconception that all 2D-Bravais lattice types are disjoint. This means that many researchers assume that there cannot be “transitions” from one Bravais lattice type to another. The concept of metric specialization [24] has, however, been created specifically to account for the “transition point” (see footnote 3) of such a transition. For situations short of a metric specialization but somewhat close to the actual transition point, one utilizes the translational pseudo-symmetry [23] concept.

If all 2D-Bravais lattice types were indeed disjoint, there would be no hierarchy among them. Also there would be no hierarchy of the plane symmetry groups [13, 17, 18, 25, 26] and no hierarchy between point symmetries in 2D [25–27]. As Fig. 1 and the quoted literature shows, all of these hierarchies do, however, exist. The square and the hexagonal Bravais lattice types are at the top of different branches of the 2D-translation symmetry hierarchy, Fig. 1, and, therefore, disjoint. A transition from one of these two Bravais lattice types to the other by means of a gradually increasing translational pseudo-symmetry is not possible.

There are actually three different hierarchy branches for the 2D-Bravais lattice types: one from the oblique (*mp*) lattice to the rectangular (primitive, *op*) lattice to the square (*tp*) lattice; another one from the oblique lattice to the rectangular centered (*oc*) lattice to the square lattice; and finally the 3rd branch from the oblique lattice to the rectangular centered lattice to the hexagonal (*hp*) lattice, see sketches in the middle of Fig. 1.

This means in the language of inferential statistics [6] that the members of each of these branches are ‘nested’ (2D-translation symmetry) models. In the language of set theory, there are inclusion relations between the translation symmetry models of the Euclidean plane which characterize the individual branches of the hierarchy. The models within a branch are said to be non-disjoint.

When one deals with nested (non-disjoint) models, one cannot simply select as preferred model the one which minimizes (Kullback–Leibler) information loss [6, 7] when it is utilized to represent data on the basis of the model’s accuracy (as measured by a suitable distance measure) alone [27]. A more general model with fewer constraints will always fit the data better than a more sophisticated model with more constraints [7, 16, 27]. A higher symmetric (more constrained) Bravais lattice type would by that logic never be selected on the basis of any pure distance measure [27].

Geometric AICs [7, 16–18] deal effectively with sets of nested (non-disjoint) models because the accuracy of each model and its sophistication/generality are both properly accounted for (as already mentioned in the “Introduction and background” section). There is also no requirement for an a priori estimate of the noise level when two non-disjoint models are compared by a G-AIC in order to find out which of the two models possesses the larger likelihood of representing the data with a minimal loss of (Kullback–Leibler) information [16, 18]. After the most likely model has been identified in a series of such pair-wise comparisons, the (Kullback–Leibler) best model is selected and its noise level is estimated (on the basis of that particular model). The probability that a certain translation symmetry type is the one with minimized information loss when it is assigned to an image can also be calculated. Akaike weights allow for predictions on the basis of a weighted average of all of the considered models [6, 18]. We will expand on this elsewhere.

It is straightforward to derive possible translational pseudo-symmetries (of the first kind) from the limiting cases of the lattice parameters of 2D-periodic arrays of points as listed in the third column of Table 1. All one needs to do is to change a single smaller than (<) or unequal (≠) sign in the second column of this table to an approximately equal (≈) sign.

**Table 1:** Aspects of the Bravais lattice types of the Euclidean plane in their crystallographic standard [25] settings (assembled from information in Refs. [17] and [29])

Bravais lattice types’ names and standard abbreviation letters [25]	Parameters of the conventional unit cells	Limiting cases of lattice parameters that lead from a lower symmetric Bravais lattice type to its higher symmetric counterpart(s), also known as metric specializations in 2D	Conventional basis vectors (*a′*, *b′*) for limiting cases [29]	Number of geometric or symmetry constraints that enter the G-AIC for the assignment of Bravais lattice types to sets of lattice parameters [17]
Hexagonal*, hp*	*a* = *b*, *γ* = 120°	None as top of a hierarchy branch is reached	None as there is no such case	*Four*, e.g., opposite sides are parallel, diagonals are orthogonal and posses ratios of $\sqrt 3$ or $\sqrt 3^{ - 1}$ (equivalent to lattice defining angle 120°)
Square*, tp*	*a* = *b*, *γ* = 90°	None as top of a hierarchy branch is reached	None as there is no such case	*Four*, e.g., opposite sides are parallel, adjacent sides are orthogonal, and diagonals are both orthogonal and of equal length (equivalent to lattice defining angle 90°)
Rectangular centered*, oc*	*a* < *b* ≠ *a*$\sqrt 3$, *γ* = 90° *For primitive sub*-*unit cell:* *a*^*♦*^ = *b*^*♦*^*, γ*^♦^ ≠ 60° or 90°	*a* = *b* → *tp* *a*$\sqrt 3$ = *b* → *hp* *For primitive sub*-*unit cell:* *γ*^♦^ = 60° → *hp* *γ*^♦^ = 90° → *tp*	(***a*** + ***b***)/2, (***b*** − ***a***)/2 in both cases *For primitive sub*-*unit cell:* ***a***^***♦***^, ***b***^***♦***^ in both cases	*Three*, e.g., opposite sides are parallel and adjacent sides are orthogonal while conventional unit cells encompass 2 lattice points *For primitive sub*-*unit cell:* opposite sides are parallel and diagonals are orthogonal but of different lengths so that their ratio is never 1, $\sqrt 3$, or $\sqrt 3^{ - 1}$
Rectangular (primitive), *op*	*a* < *b*, *γ* = 90°	*a* = *b* → *tp*	***a***, ***b***	*Three*, e.g., opposite sides are parallel and adjacent sides are orthogonal
Oblique*, mp*	− 2*b* cos *γ* < *a* < *b*, 90° < *γ*	*γ* = 90° → *op* − 2*b* cos *γ* = *a* → *oc* *a* = *b* → *oc*	***a***, ***b*** ***a***, 2***b*** + ***a*** (*a′*$\sqrt 3$ < *b′*) ***a*** + ***b***, ***b*** **−** ***a*** (*a′*$\sqrt 3$ > *b′*)	*Two*, e.g., (two) opposite sides are parallel

This setting arises from a projection through a crystal along its third dimension and is employing a right-handed coordinate system so that the ***c*** (or ***z***) axis vector points into the page away from the reader. Vectors are given in bold face font and their magnitudes are in an ordinary font. The third column of this table lists the limiting cases (physical degeneracies) that are pertinent to the metric hierarchy of the Bravais lattice types as shown graphically in the middle-left sketch of Fig. 1. The ^♦^ sign refers to the primitive sublattice of the rectangular centered Bravais lattice

For example, if lattice vector magnitudes *a*^♦^ and *b*^♦^ were extracted from a more or less 2D-periodic image with error bars ± Δ*a*^♦^ and ± Δ*b*^♦^ and the angle *γ*^♦^ between the corresponding vectors ***a***^**♦**^ and ***b***^**♦**^ was extracted with an error bar of ± Δ*γ*^♦^ so that *b*^♦^ − Δ*b*^♦^ ≤ *a*^♦^ ± Δ*a*^♦^ ≤ *b*^♦^ + Δ*b*^♦^ (or *a*^♦^ ≈ *b*^♦^ within error bars, in other words) and the interval *γ*^♦^ ± Δ*γ*^♦^ contains the 60° value, an ambiguity arises if one is dealing with an oblique Bravais lattice, or the primitive sub-unit of a rectangular centered Bravais lattice, or a hexagonal Bravais lattice. This ambiguity is due to a translational pseudo-symmetry. As shown in the sketch in the middle-right of Fig. 1, the ^♦^ signs (that we used above) signify parameters of the primitive sublattice of a rectangular centered (*oc*) Bravais lattice.

The same example can also be discussed on the basis of the conventional (i.e., centered) unit cell parameters of the *oc* Bravais lattice type. For this, the interval *b* ± Δ*b* needs to contain the value $a\sqrt 3$ ± Δ*a* (or $a\sqrt 3$ ≈ *b* in other words), while the angle *γ* between the conventional [10] and [01] vectors of this lattice needs to be within an error bar that contains the 90° value.

Reducing the widths of the error bars on the extracted lattice parameters of this example sufficiently (by, e.g., a more accurate extraction that is aided by a lower noise level) so that the approximately equal (≈) sign between the lattice vector magnitudes can be safely ruled out and/or the 60° value is excluded from the extracted lattice angle interval would be one way to deal with this ambiguity. Utilizing a G-AIC and Akaike weights [18] for the classification of the lattice parameter set of the preceding paragraph would, on the other hand, result for the original error bars in model probabilities larger than zero for all three members of the Bravais lattice type hierarchy branch *mp* → *oc* → *hp*, see Fig. 1. Between these three hierarchy branch members, one could identify one Bravais lattice type as being the most likely translation symmetry type given the nested geometric model set (*mp*, *oc*, and *hp*), the noisy data, and the set of corresponding maximal likelihood lattice parameter extraction results. Future data with a lower noise level could result in one of the other two models in the set being the most likely translation symmetry. We will expand on this elsewhere.

When error bars are zero, i.e., in a mathematically strict and abstract sense, the conceptual basis for the above discussed translational pseudo-symmetries disappears. Correspondingly, approximately equal (≈) signs in the relations between the lattice parameters would no longer be allowed.

Limiting cases for lattice parameters that lead in the abstract mathematical sense from a lower symmetric Bravais lattice type to its higher symmetric counterpart(s) are listed in column 3 of Table 1. Note that the approximately equal (≈) signs of the previous paragraph are in the limiting cases replaced by strictly equal (=) signs. A collective term for the limiting cases in Table 1 is metric specialization [24] in two dimensions.

The number of limiting cases that lead to higher symmetric types of Bravais lattices in Table 1 is also the number of upward leading lines between nodes in the combined 2D-translation/plane symmetry hierarchy sketches in the middle of Fig. 1, i.e., two for *mp*, one for *op* and two for *oc*. It is also clear from the fourth column of Table 1 that the area of the primitive sub-unit cell, which contains one lattice point, is doubled when one uses the conventional *oc* Bravais lattice setting (which encompasses two lattice points). This is because the *oc* limiting case of the oblique (*mp*) Bravais lattice type possesses twice the area of the oblique unit cell. Correspondingly, the *hp* limiting case of the (*oc*) rectangular centered Bravais lattice type possesses only one half of the rectangular centered unit cell area. Alternative translation vectors need, therefore, to be chosen when one “moves up” in the *mp* → *oc* → *hp* hierarchy branch, see middle sketches in Fig. 1.

As is very well known, Fourier transforms relate corresponding pieces of information in direct and reciprocal space to each other per mathematically defined relations. The intertwined symmetry and metric properties of a Bravais lattice type are, as a consequence, independent of the space in which one chooses to work [17, 18]. It is, therefore, straightforward to convert the direct space unit cell shapes that are sketched on the middle-right hand side of Fig. 1 into representations of reciprocal space unit cells. All one needs to do in this case is to change annotations and shapes so that they no longer refer to the direct space, e.g., change *γ* = 120° to γ* = 60° (where the * sign stands for the reciprocal space).

In the amplitude map of the dFT of a 2D-periodic image with one of the plane symmetries that is compatible with the rectangular centered Bravais lattice type, i.e.,* cm* or *c2mm*, one needs to label the ‘diffraction peaks’ in a way that index sums are always even because one considers all odd integer sum ‘reflections’ as being systematically absent [25]. As a matter of fact, no Fourier coefficient (FC)/reflection is actually (physically) absent. We just have to consider all odd index sum reflections in 2D as possessing zero amplitude in order to obtain a rectangular centered reciprocal unit cell of one half of the area of its primitive sub-unit cell so that we have a doubling of the primitive sub-unit cell area in direct space [17].

In other words that may be more appealing to materials scientists and electron microscopists, the shortest vectors that are present in the [001] oriented transmission electron diffraction pattern of a very thin cubic body-centered crystal (such as tungsten) are of the {110} type, i.e., *h* + *k* + *l* = even. Analogously, vectors of the type {11} are shortest in the amplitude map of a dFT of a 2D-periodic array that possesses the rectangular centered Bravais lattice type. The vectors (*h*0), (0*k*) and (*hk*), where *h* = odd, *k* = odd, and *h* + *k* = odd, are all considered to have zero amplitude. The first non-zero amplitude Fourier coefficients (FCs) along the 〈10〉* and 〈01〉* directions (in reciprocal space) are then labeled as (20) and (02). (For this little example, it was completely immaterial that we used analogies between mathematically abstract and physically real concepts as well as spaces of either 2 or 3 dimensions).

This is all very different from systematic absences that are due to glide lines in 2D (as well as glide planes and screw axes in 3D), where certain “odd reflections” obtain zero amplitude by destructive wave interference in single scattering experiments or correspondingly by mathematical superpositions of complex-number valued FCs [25]. These reflections or FCs are actually (physically/mathematically) absent or genuinely extinct in other words.

Since the point/space symmetry and metric properties of the Bravais lattice types are intertwined, programs such as CrysTBox and CRISP that display dFT amplitude maps of more or less 2D-periodic images from which they extract lattice parameters allow the user to assess the 2D-Laue symmetry class of the images visually. This kind of symmetry is based on the amplitudes of the FCs around the central (00) peak, rather than their positions in this map. The Laue classes in 2D are the six point symmetry groups *2*, *2mm*, *4*, *4mm*, *6* and *6mm*. Four of these point symmetries, i.e., *2*, *2mm*, *4mm*, and *6mm*, are holohedries and, therefore, in 2D responsible for the one to one correspondences between the lattice systems and crystal systems.

References [25, 26] provide good introductions to geometric-structural crystallography, e.g., the intertwined nature of the metric relations and plane symmetries that characterize the Bravais lattice types. The former of these two references is the brief teaching edition [25] of the most definitive text on this subject, i.e., volume A of the International Tables for Crystallography (which has been extensively revised and updated in 2016 in its 6th edition), and the latter is a good college level textbook with emphasis on crystallography in 2D [26].

## Overview I: synthetic test image sets

All of the 12 synthetic 2D-periodic images used for our lattice parameter extraction review are presented in Fig. 2. Eight of these images, i.e., #1, 3, 4, 6, 7, 9, 10, and 12, were also shown and analyzed in Ref. [19]. Two of these images, i.e., #7 and #8 were also discussed in Ref. [18]. As mentioned in the abstract, there is per design one noise-free (i.e., strictly 2D periodic) image and two noisy (i.e., more or less 2D periodic) images in each of the four sets of three images. These sets are arranged in columns in Fig. 2.

The noise-free images are at the top of each of these columns in the first row of Fig. 2. Note that according to Kenichi Kanatani’s dictum [16], different geometric feature extraction algorithms will obtain, even for these four images, slightly different results in a systematic manner. This must be so because different heuristics that include internally defined thresholds and parameters as well as approximations are embedded in different algorithms. One may think of this loosely as the different algorithms themselves introducing some small systematic errors (or feature extraction uncertainties) into the lattice parameter extraction results, which are more or less specific to the processed image [16].

The second row of this figure consists of images where independent Gaussian noise of mean zero and a standard deviation of 10% of the maximal pixel intensity was added to the individual pixels. The third row in Fig. 2 finally provides the test images to which independent Gaussian noise of mean zero and a standard deviation of 50% of the maximal pixel intensity was added.

One may consider the 10% amount of added noise to be “small to moderate” relative to the signal in the images because the latter is highly redundant due to its 2D-periodic nature. The 50% added noise may then be considered as “moderate to excessive” with respect to the signal in the images for the same reason.

Of all of the test images, the first two in the second column, i.e., images #4 and # 5, should present the least challenge to any lattice parameter extraction algorithm because there is a clear difference in the magnitudes of the two lattice vectors and a 90° lattice angle per design while additional noise is either non-existent or small to moderate. Also these two images are composed of approximately 175 “sub-images” of individual unit cells so that an effective averaging can take place by suitable algorithms to reduce the effective noise level of the average unit cell.

There are also no genuine pseudo-symmetries per design in the noise-free image #4. Note, however, that all symmetries in image #5 are only pseudo-symmetries of the second kind because all originally existing symmetries were unavoidably broken by the addition of 10% Gaussian noise. (No pseudo-symmetry of the first kind was introduced into image #5 per design so that all pseudo-symmetries in this image originate from the noise-induced breaking of the symmetries that are present in image #4).

All of the images in Fig. 2 are *calculated* images in Vasco Ronchi’s sense rather than experimentally detected images [41]. The point spread function of the imaging instrument is assumed to be exactly known in calculated images so that an image can be described with unlimited precision by a perfectly fitting mathematical model. In the case of calculated noise-free images that are perfectly periodic in 2D, these models are the plane symmetry groups [25]. Detected images will always be noisy and the prevailing point spread function of the detection apparatus will never be exactly known [41]. Experimentally detected images will, therefore, never *really* possess Bravais lattices and plane symmetries because both concepts are mathematical idealizations. On the other hand, it makes a lot of sense to assign Bravais lattices, Laue classes, and plane symmetries to detected images from crystals that are reasonably periodic in 2D because a very large reduction of the dimensionality of the model parameter space is obtained by such approximations.

We consider the independent Gaussian noise of mean zero that has been added to the images of the top row of Fig. 2 in order to create the image pairs #2 #3, #5 #6, #8 #9, and #11 #12 as a reasonable equivalent to random errors of a hypothetical imaging process [16] by which these images could have been detected from strictly regular 2D-periodic arrays of points of variable sizes and intensities. From the design history of all of the test images, it is clear that there are no systematic errors in either the translation symmetries or the site symmetries within all unit cells throughout all of the synthetic test images.

Throughout the remainder of this paper, we follow the 2D lattice setting of the CRISP program [21] as valid alternative to the crystallographic standard settings [25] of Table 1. The direct space unit vector ***a*** (or ***x***) points in all of the images of this paper from the left to the right horizontally (when read into the CRISP program) and the unit vector ***b*** (or ***y***) is directed vertically upwards.

For a right-handed coordinate system in 3D, this corresponds to a ***c*** (or ***z***) vector which points out of the paper towards the reader. As one can appreciate visually in the first three columns of Fig. 2, this alternative setting of 2D lattices leads, most of the time, to lattice vector magnitude relationships of the type *b* < *a*, but retains the *γ* > 90° condition (of Table 1) for the oblique Bravais lattice type. (While in formal disagreement with some of the entries in Table 1 in an utterly non-essential way, one is free to choose the settings of *mp*, *op* and *oc* Bravais lattices as one pleases.)

The three images in the first column in Fig. 2, possess per design an oblique Bravais lattice with a *b*/*a* ratio of approximately 1.0018 and a lattice angle of 90° + arctan (^3^/_50_). By standard crystallographic convention [25], see also Fig. 1, this Bravais lattice type is abbreviated with the letter combination *mp* in Tables 3, 4, 5 and 6 below. To the human eye, the horizontal rows of dots appear to be identical in image #1 while there are actually very slight intensity differences that are periodic in every second row. Arrangements such as this are technically analogous to superlattices that arise from atomic ordering in mixed crystals. It would, however, be too much to ask of any of the tested lattice parameter extraction algorithms/programs to pick up this miniscule variation of intensities so that an experimental *b*/*a* ratio of approximately one half will be the expected result. When noise is added to image #1 in order to produce images #2 and #3, the very tiny intensity differences of subsequent horizontal rows of the noise-free image are “washed out” so that the features of a superlattice are hidden.

Also the plane symmetry of image #1 is *p1* per design, i.e., the only site symmetry of the 2D-periodic motif is the identity (360° rotation) operation. Groups of three white dots in this image are related to each other by broken twofold rotation points so that this image features a rather strong motif-based pseudo-symmetry of the first kind, or in other words, an intentionally (per design) broken *p2* plane symmetry. (A quantitative measure for this pseudo-symmetry is an average Fourier coefficient phase angle deviation from 0° or 180°, which has been determined with CRISP in its default setting to be just 5.7°).

Added independent Gaussian noise of mean zero is bound to either exacerbate (as perhaps in images #2) or diminish (as perhaps in image #3) this pseudo-symmetry of the first kind. Since plane symmetry *p2* is holohedric, it is also the plane symmetry of the oblique Bravais lattice type so that this motif-based pseudo-symmetry (of the first kind) does not present a challenge to lattice parameter extractions algorithms.

The six images in the second and third columns in Fig. 2 possess per design rectangular primitive (*op*) Bravais lattices. Clearly discernible to the unaided human eye are intensity differences in the set of three horizontal dots in image #7 at the top of the 3rd column of Fig. 2 so that one would assign an *op* Bravais lattice type to this image by visual inspection. The other two images of this test set (#8 and #9) possess obviously per design that same translation symmetry type.

With the intensity differences of the set of dots, i.e., a major part of the translation periodic motif of this image, somewhat “washed out” in the latter two images, an assignment of the qualitatively correct (*op*) Bravais lattice type to these two images by visual inspection becomes difficult. This is especially true for the image with the largest amount of added noise (#9). The difficulty is due to a combination of the per design existing pseudo-symmetries (of the first kind, both motif-based and translational as in image #7) with the added Gaussian noise in these two images of this test image set. In other words, the per design existing genuine symmetries (that form plane symmetry group *pm* as a crystallographically allowed combination of genuine site and translation symmetries) are turned into pseudo-symmetries of the second kind by the addition of the noise and it is the combination of both kinds of pseudo-symmetries that presents the challenge to assigning a qualitatively correct Bravais lattice type by visual inspection to images #8 and #9. Apparently, a broken *4mm* point symmetry (and corresponding Laue class in Fourier space) arises from the “washing out” of the intensity differences of the three dots in the translation periodic motif of image #7 so that the lattice constant in the horizontal direction is reduced to approximately one third of the true lattice constant. The large amount of independent Gaussian noise in image #9 exacerbated the tendency that is already noticeable in image #8.

The fourth column of Fig. 2 shows three images (#10 to #12) that possess a translation periodic motif that requires a rectangular centered (*oc*) Bravais lattice. Images #11 and #12 show extreme cases of a translational pseudo-symmetry of the special (see footnote 2) kind. This is because of the fact that the noise-free image of this set (#10) possesses per design a metric specialization (see footnote 3) at the primitive sublattice (*γ*^♦^ = 90°, *a*^♦^/*b*^♦^ = 1) level. The conventional (centered) lattice possesses consequently also a metric specialization (*γ* = 90°, $\sqrt 2$*a*^♦^/$\sqrt 2$*b*^♦^ = 1).

The primitive sub-units of the lattices of images #10 to #12 are per design a perfect square with edges of 12 × $\sqrt 2$ pixels. There are, however, no fourfold rotation points in the translation periodic motif of these three images that a “genuine [non-detached (see footnote 3)] crystallographic” square lattice would require. This is most clearly seen in the noise-free image of this test set (#10) for the obvious reason that no noise obscures the design (and that there are, therefore, no pseudo-symmetries of the second kind).

The distance ratios of the nearest neighbors of all white dots in image #10 are either unity or $\sqrt {45}$/6 and support both point symmetry (Laue class) *2mm* and the lattice centering translation. For the purposes of this review, two synthetic images that represent extreme cases of translational pseudo-symmetry [of the special (see footnote 2) kind] due to adding Gaussian noise to a synthetic (noise-free) image with metric specialization (see footnote 3) suffice. (In Ref. [19], the corresponding set of images is referred to as “hex lattice with vacancies”, but there are neither three- or six-fold rotation points nor vacancies).

When the lattice angle that has been extracted from a noisy image is (in direct space) close to 90° or 120° (within error estimates) and the magnitudes of the unit cell vectors are close to being equal (within error estimates), many researchers would not consider the possible existence of a rectangular centered Bravais lattice where the *γ*^♦^ angle can per definition be neither 90° nor 60°.

This is because it somehow seems “more natural” to assume that there would neither be a pronounced translational pseudo-symmetry of the first kind nor a metric specialization with an associated translational pseudo-symmetry of the special kind. Instead many researchers would conclude that these lattice parameters are compatible with either the square (*tp*) or hexagonal (*hp*) Bravais lattice types, if the error bars allow for these conclusions. There is, however, no objective basis to rule out lower symmetric Bravais lattice types to justify these conclusions either by a human being or by a currently existing computer program. The Committee on Statistical Descriptors of the International Union of Crystallography was well aware of this fact when it stated that *“Thoughtless use of established procedures in widely distributed software may be as harmful as the natural tendency of most people to prefer results in agreement with preconceived ideas.”* [42, 43].

As a whole, the test images of this review are ideal as objects to assess the performance and robustness of the three algorithms/programs on both noise-free and noisy images. As a matter of fact, one may consider the calculated test images to be reasonable equivalents of images that have been recorded at different signal-to-noise ratios with a “perfect microscope” where the microscope’s point spread function is the Dirac delta function.

The calculated noisy test images are also suitable for objective (i.e., arbitrarily set threshold free) G-AIC-based classifications [7, 16] of their Bravais lattice types on the basis of maximal likelihood extracted lattice parameters [17, 18] because systematic errors (that are unavoidably introduced by the applications of the algorithms) should be small compared to random errors that are caused by the added Gaussian noise. As already mentioned above, we will report on these classifications elsewhere.

As the 2D-lattice parameters of images #1 to #12 are known per design, one could make an assessment of the accuracy with which the three tested computer programs extract these parameters on the basis of their a priori known values. (In the computational symmetry, remote sensing, and computer vision/robotics communities, these kinds of a priori known values are referred to as the “ground truths”.) While we will do this elsewhere, below we will use reasonable estimates for error bars on the extracted lattice parameters and calculated geometric quantities such as the *b/a* ratios and the unit cell areas that are obtained directly from the outputs of the employed programs.

This approach allows for an assessment of the presumed accuracy and precision of the three tested programs on the basis of their outputs alone, i.e., independently of the known quantitative design parameters of the synthetic test images, and will lead us to conclusions on which kinds of precisions are typically obtainable for the task at hand. We will, however, use our knowledge of the a priori known Bravais lattice types that are assigned to the images per design in our discussions.

## Overview II: tested algorithms/computer programs

The first of the three algorithms/programs that we tested extracts the parameters of primitive 2D lattices in direct space [19]. The other two programs utilize reciprocal (Fourier) space for the extraction of lattice parameters [20, 21] so that they possess the advantage of averaging over the periodic direct space information effectively as a byproduct. They are, therefore, both expected to perform better in the presence of noise than the first algorithm/program.

Note that we did not make a clear distinction between an algorithm and a computer program in the preceding paragraph because that is irrelevant to the main thrust of this paper. As already stated in the introduction, this thrust is to illustrate Kenichi Kanatani’s dictum that there are *no* definitive geometric feature extraction algorithms in all real-world applications [7, 16] and, therefore, also no definitive extraction results in real-world imaging experiments that could be utilized for a subsequent qualitatively *definitive* crystallographic classification of these results, such as the assignment of a 2D-Bravais lattice type.

A good computer program for the extraction of lattice parameters from more or less 2D-periodic images is an implementation of a suitable algorithm for the task at hand. All three of the tested programs fall into this category as Refs. [12, 19–21] (and the approximately 300 citations on Elsevier’s Science Direct website for Ref. [21]) attest. As will be illustrated below in the following section, the lattice parameter extraction results of all three computer programs/algorithms are nevertheless *not definitive*. The reasons that this must be so are provided in Refs. [7, 16].

For all three of the tested computer programs, it is up to the user to classify the extracted lattice parameters as belonging to one of the five types of translation symmetries, i.e., 2D-Bravais lattice types, which exist per crystallographic convention [25] in the Euclidean plane. This includes also decisions as to whether or not the image data are compatible with a centered unit cell so that the image is to be classified as featuring the rectangular centered Bravais lattice type. Table 2 gives a brief overview over the employed three computer programs and the algorithms behind them.

**Table 2:** Overview of the three programs/algorithms that were employed in this review

Program name	Primitive Unit Cell Extraction (PUCE), version 2.0	CrysTBox, server 1.08 (build 0039)	CRISP, version is 2.1
References	[19]	[20]	[21] refers to the first version of the program
Year of the publication that introduces the program	2015	2015	1992
Operating system to run the program	Unix/Linux and all usual platforms, needs to be compiled by the user	Microsoft Windows, 32 and 64 bit versions (up to version 10)	Microsoft Windows (up to version 10)
Space of lattice parameter extraction	Direct	Reciprocal/Fourier	Reciprocal/Fourier
Availability	Directly from https://github.com/nmevenkamp/UnitCellExtraction for free	Directly from the program’s website: http://www.fzu.cz/en/crystbox for free after registration	Commercial, see http://www.calidris-em.com/crisp.php
Types of input files	Single (16 bit) and double precision (32 bit) *.tif	Many usually encountered file formats of images, e.g., *.tif, *.tiff, *.bmp, *.png, *.jpg, as well as *.dm3 and *.dm4 (i.e., DigitalMicrograph/Gatan specific image formats)	*.jpg and 8-bit baseline feature (standard) *.tif
Distinctive features of the algorithms	Traditional AIC-based model selection [5] for the most likely translation symmetry/primitive unit cell; only deals with single crystal images	Based on concepts from the computer vision and robotics fields, e.g., differences of Gaussian filters in Fourier space and the so-called RANSAC algorithm for the assignment of the reciprocal lattice [12] Can deal with electron diffraction patterns and images that contain information from at least two different crystal phases, e.g., from a crystalline inclusion within a crystalline matrix Mainly designed for electron diffraction work, but analysis of more or less 2D-periodic images in Fourier space is also supported; sliding Fourier transform window applications on the basis of this program for the mapping of structural inhomogeneities [12]	Rewritten code on the basis of Fortran code that constitutes the electron crystallography program suite of the Medical Research Council of the University of Cambridge, U.K., see http://www2.mrc-lmb.cam.ac.uk/research/locally-developed-software/image-processing-software/, which was developed over the last five decades Only deals with single crystal images
Programming language	Written in C++	Implemented in MATLAB, also as plugin for Gatan’s DigitalMicrograph	Written in C with a few subroutines in assembler

The CRISP program [21] is the only one of these three computer programs that allows also for systematic assessments of possibly existing pseudo-symmetries of the first and special kinds. This is because, in addition to extracting the lattice parameters from the intensity distribution in a noisy (i.e., more or less) 2D-periodic image, the CRISP program outputs allow for the (somewhat subjective) determination of its Laue class and plane symmetry group as part of its electron crystallography [44] support functionality. The inherent subjectivity of all of these determinations could be overcome by using G-AICs and Akaike weights [18], but no suitable computer programs are yet available for these kinds of tasks.

For noise-free images that also do not contain distortions (such as the ones shown in the first row of Fig. 2), plane symmetry detection is trivial [25] and one can assign a perfectly fitting plane symmetry group directly by visual inspection. As all site and translation symmetries are broken by added Gaussian noise, one can, on the other hand, only derive the most likely plane symmetry and Bravais lattice type from noisy images by objective methods such as G-AICs [7, 16–18] which do not utilize arbitrarily set thresholds. We will report on the determination of the Laue classes and plane symmetries of all test images of Fig. 2 elsewhere.

With the CrysTBox program [21], one has at least visual access to the dFT amplitude map of more or less 2D-periodic images so that one may notice when the point symmetry of this map (i.e., its Laue class) is in qualitative disagreement with the translation symmetry type that one would infer from of the extracted lattice parameters and their error bars. This helps in detecting pseudo-symmetries of the first and special kinds.

Also the visual dFT amplitude maps that the CrysTBox program outputs are useful for assessing whether or not the numerical outputs of the lattice vector magnitudes refer to the two shortest reciprocal lattice vectors. These kinds of assessments are necessary because the CrysTBox program follows the strategy to assign the reciprocal lattice basis vectors to FCs with large amplitudes, which are not necessarily also the two shortest reciprocal lattice vectors. When the numerical outputs of this program do not include information on the two shortest reciprocal lattice vectors that are visible in the dFT, one needs to obtain qualitatively correct lattice parameters by re-calculating them from the provided numerical outputs of the CrysTBox program.

No prior information on the unit cell parameters of the “crystalline materials” was used as inputs for the CrysTBox and CRISP programs so that they would extract lattice parameters just from the geometric information in the images (and could not be aided in any conceivable way by their inbuilt databases). This disables error estimations in CrysTBox on the basis of the comparison of extracted lattice parameters with their theoretical counterpart for a known crystalline material, magnification, and microscope calibration. (As we are concerned in this review with the extraction of lattice parameters from more or less 2D-periodic images of “unknown materials”, this disablement is of no further consequence to us.)

Neither the Primitive Unit Cell Extraction (PUCE) program [19] nor the CrysTBox program [20] is designed to extract lattice parameters that correspond to rectangular centered Bravais lattices. The CRISP program [21], on the other hand, possesses this functionality.

For the PUCE program, there are no alternative settings or options. A small program was written for a python interpreter to prepare the lattice parameter extraction results of the PUCE program for listings in Tables 3 and 5 below. This program also calculates the error estimates for these listings for variable choices of error estimates for this program’s numerical output and is available on request from the second author of this paper.

In case of the CrysTBox program, there are output windows for the magnitudes of the direct and reciprocal lattice vectors as well as for the magnitude ratios of four reciprocal lattice vectors that the program identified. One needs to read off the angles between the individual FCs in the amplitude map of the dFT that this program outputs and add them up in order to obtain the reciprocal (and direct space) lattice angle parameters. The CRISP program provides result output windows where one can read off the reciprocal and direct space lattice parameters directly.

It has been reported that the PUCE program performs well for images with reasonably small amounts of Gaussian noise [19]. Note that it is explicitly stated in Refs. [12, 19, 20] that the outputs of the CrysTBox and PUCE programs are highly accurate and precise. In the case of the PUCE program, sub-pixel precisions are stated (at least for all of the noise-free images) for extracted Cartesian coordinates from which the lattice parameters are to be derived [19]. This results for the synthetic test images of this review in relative errors on lattice vector magnitudes of a few percent.

Analyses of two experimental HRTEM images are mentioned in Ref. [20] as examples where reciprocal lattice vector magnitudes as extracted with CrysTBox are compared to their theoretical reference values. The extraction results agreed with the theoretical reference values to better than 1%, on average, and were slightly more accurate than the lattice parameter magnitudes that two experienced human analysts derive from the same images by other means. Reference [12] reports an accuracy of approximately 0.1% for lattice vector magnitudes that were extracted with the CrysTBox program so that translation periodicity deviations in an epitaxial deposit could be quantified in a cross section of a HRTEM sample.

Because sufficiently accurate FC phase angles can be extracted by CRISP as part of its electron crystallography [44] support functionality, reciprocal lattice vector magnitudes must be extracted with a precision of better than one half of a pixel (in reciprocal space) for even the highest diffraction orders [21]. This requirement is fulfilled by two least-squares refinement cycles for the assigned reciprocal lattice.

None of the tested programs provide estimated error bars on their outputs in an explicit form (when no theoretical reference lattice parameters were entered into the inbuilt database of CrysTBox, as mentioned above). The CRISP program outputs direct lattice vector magnitudes with three digits. The extracted direct space lattice angles outputs of this program comprise three digits in case of angles smaller than 100° and four digits otherwise.

The other two programs output their results with significantly more digits, which we rounded to the same presumed order of magnitude accuracy as the results of the CRISP program for displays in Tables 3, 4, 5 and 6.

More specifically, the PUCE program outputs Cartesian coordinate pairs for the two extracted direct space lattice parameters as 32-bit floating point values. From these values, the lattice vector magnitudes, their ratio, the lattice angle, and the unit cell area were calculated (in direct space) with the above-mentioned small python program (that is available from the second author of this review on request).

The CrysTBox program delivers 5 to 6 digit outputs for the magnitudes of direct space lattice vectors and rounds the corresponding reciprocal space lattice vectors to 5 digits after the decimal point. For the reciprocal lattice angle, this program delivers four digit outputs including trailing zeros.

All three tested computer programs should, in summary, extract lattice parameters with a high accuracy and precision while being based on different algorithms. The CrysTBox program is dedicated to analyses of known crystalline materials on the basis of electron diffraction patterns and offers a Fourier transform route to the processing of more or less 2D-periodic images (of known and unknown origin) as a sideline. The CRISP program, on the other hand, is dedicated to crystallographic image processing [13] and electron crystallography [44], but also offers complementing analyses of electron diffraction spot patterns by an extension module.

An *ideal* geometric feature extraction algorithm would provide unbiased (accurate) results when applied to a calculated image. This means that no systematic error would be introduced into the extraction results by the algorithm itself. The algorithm would also work for any level of complexity of the input images. Pre-existing systematic errors in synthetic images would be faithfully propagated by such an algorithm to the geometric-structural feature extraction results along with the faithful propagation of the consequences of the noise in the images. Due to calculations with real numbers of finite length as floating point representations (including 64-bit double-precision numbers of the IEEE 754-2008 standard), subsequent rounding and calculation errors, utilized approximations and heuristics, computer programs that implement geometric feature extraction algorithms can at best come close to this ideal [16].

Because we tested three 2D-lattice parameter extraction computer programs on *calculated* images that do not contain systematic errors by themselves, essentially only random errors should have propagated to the extraction results if the corresponding algorithm implementations were close to the *ideal* algorithm implementation of the preceding paragraph. If the three computer programs/algorithms that we applied to the images in Fig. 2 were indeed close to this ideal, we should have obtained essentially the same lattice parameter extraction results for all three of them, whereby the widths of the error bar intervals could have varied somewhat.

## Particulars of the employed lattice parameter extraction procedures

The default^9^ settings of the two programs/algorithms that extract lattice parameters in reciprocal/Fourier space [20, 21] were used in parts of this review and the corresponding results are reported in Tables 3 and 5. For the calculations of dFTs with the CRISP program, we also selected the maximal circular area of the images (i.e., a disk with a diameter of 256 pixels) as an alternative (non-default) setting for the least-squares extraction of lattice parameters. The corresponding results are reported separately in Tables 4 and 6.

Informed by our previous work with the CRISP program [13], we utilized the manual reciprocal basis vector assignment option whenever the automatically (by default) assigned reciprocal lattice in the dFT amplitude map was obviously incorrect by visual inspection. This could, for example, be due to a translational pseudo-symmetry of the first or special kind. Similarly, we also made inferences from the visual inspection of the apparent point symmetry in the amplitude map of the dFT, i.e., the apparent Laue class, of an image concerning the possible existence of a motif-based pseudo-symmetry of the first kind or of a metric specialization that has been turned into a translational pseudo-symmetry of the special kind by noise in that image.

Whenever lattice vectors of the same magnitude were extracted with the CRISP program within reasonable error estimates, we extracted in addition to the primitive lattice parameters also the parameters of a possibly existing rectangular centered Bravais lattice by using the corresponding alternative program setting. With the CRISP program, this amounted to just one extra click with the computer mouse (and its consequences). The existence of a rectangular centered Bravais lattice was then either confirmed or rejected on the basis of the traditional plane symmetry deviation quantifiers [13, 17, 44] that CRISP delivered for both a primitive unit cell in the default setting and a centered unit cell in the alternative setting.

When the CrysTBox program [21] extracted lattice parameters from images for which we inferred the presence of a translational pseudo-symmetry of the special kind or of a metric specialization on the basis of the visual inspection of the point symmetry in their dFT amplitude maps, we used the program’s outputs for the calculation of the lattice parameters of an alternatively existing rectangular centered Bravais lattice by hand. Re-calculations of the outputs of the CrysTBox program were actually necessary in the majority of cases as will be discussed in detail in the following section. The corresponding results are reported separately in Tables 4 and 6.

Also, the visual dFT amplitude maps that the CrysTBox program outputs proved to be very useful for assessments if the numerical outputs of the lattice vector magnitudes refer to the two shortest reciprocal lattice vectors. Indeed, for images #1 to #3 and #6 to #9, i.e., more than half of the images in Fig. 2, we needed to re-evaluate/re-calculate the lattice parameter outputs of this program on the basis of the available visual outputs of the dFTs of these images.

As we mentioned already above, the PUCE program only extracts primitive lattice parameters per design and does not provide any indication that one may have actually extracted the parameters of the primitive sublattice of a rectangular centered Bravais lattice or if there might be a pseudo-symmetry of the first or special kind. As there are also no options in this program, our lattice parameter extractions with this program were limited to making inputs and receiving straightforward outputs. The corresponding results are reported in Tables 3 and 5.

Our approach to estimating the accuracy and precision of lattice parameter extraction results treats noise-free and noisy test images as if they originated from the (zone-axis projection) imaging of some unknown crystals for which only the projected Bravais lattice types but not the actual lattice parameters are known. In the "Results and discussions" section below, we will, therefore, refrain from absolute statements about whether or not extracted lattice parameters and results that were derived from them are correct in a definitive (quantitative) sense. In order to comment on these kinds of results, we will instead revert to qualitative likelihood statements. For example, when two or even all three of the tested algorithms deliver essentially the same result within reasonable error bars for an image, they are to be considered as correct in a qualitative sense with a high likelihood.

Our assignments of higher symmetric Bravais lattice types, i.e., higher than oblique, to noisy images are not *definitive* because we made the point repeatedly above that one cannot, as a matter of principle, do such qualitative assignments with certainty on the basis of the numerical values of the extracted lattice parameters and their error estimates in all real-world applications. Only when the lattice parameter extraction results required the assignment of an oblique Bravais lattice type, we did so and consider this as definitive because the corresponding translations symmetry is at the bottom of the translation symmetry hierarchy as discussed in the “Bravais lattice types in two dimensions” section. Also, in these cases, there were no doubts at all that a higher symmetric Bravais lattice type cannot be present due to its exclusion by the error estimates on the extracted lattice parameters.

Assignments of the oblique Bravais lattice type (and all higher symmetric types) require that there is genuine translation symmetry present in an image. One could argue that this cannot be the case, as a matter of principle, when noise is also present in an image. We take here the pragmatic position that approximate translation symmetry suffices for making translation symmetry type classifications feasible for real-world images.

## Results and discussions

Besides the fact that all three computer programs/algorithms aimed at an intrinsically impossible task [7, 16] in a real-world application when they extracted 2D-lattice parameters from the same sets of synthetic test images, one would naively expect that they still provide similar results in their *default* settings and *without* a re-interpretation/re-calculation that is indicated to be necessary by a program’s output such as the amplitude map of the dFT of an image. As Tables 3 and 5 show, this is often not the case.

Results that were obtained with a non-default setting of the CRISP program (e.g., mainly one extra click with the computer mouse to select the largest possible circular area of the image for subsequent processing), are listed separately in Tables 4 and 6. In these two tables, there are also results from the CrysTBox program that were re-interpreted/re-calculated. (As mentioned above in the “Particulars of the employed lattice parameter extraction procedures” section, such re-interpretations and re-calculations were indicated as being necessary after inspections of the amplitude maps of the dFTs of the corresponding test images.) While both sets of tables have the same general outlay, there are only results from CrysTBox and CRISP in Tables 4 and 6 since the PUCE program possesses only one (default) setting and does not provide amplitude maps of the dFTs of the test images on which one could base re-interpretations and re-calculations.

The agreements between the corresponding entries for the extracted lattice parameters in Tables 4 and 6 are much better than for their counterparts in Tables 3 and 5 (where only default settings have been used and no re-interpretations/re-calculations have been undertaken). As there are no genuine error bars given on any of the outputs by any of the three programs we tested, we cannot elucidate how exactly error bar interval widths on the extracted lattice parameters correlate with the amount of Gaussian noise in the synthetic test images. In a qualitative way, there is obviously such a correlation in Tables 3, 4, 5 and 6.

**Table 3:**
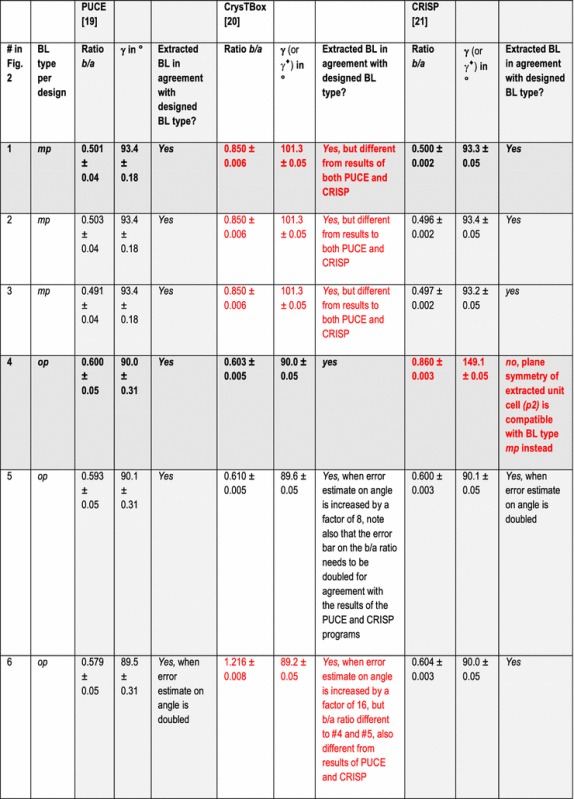

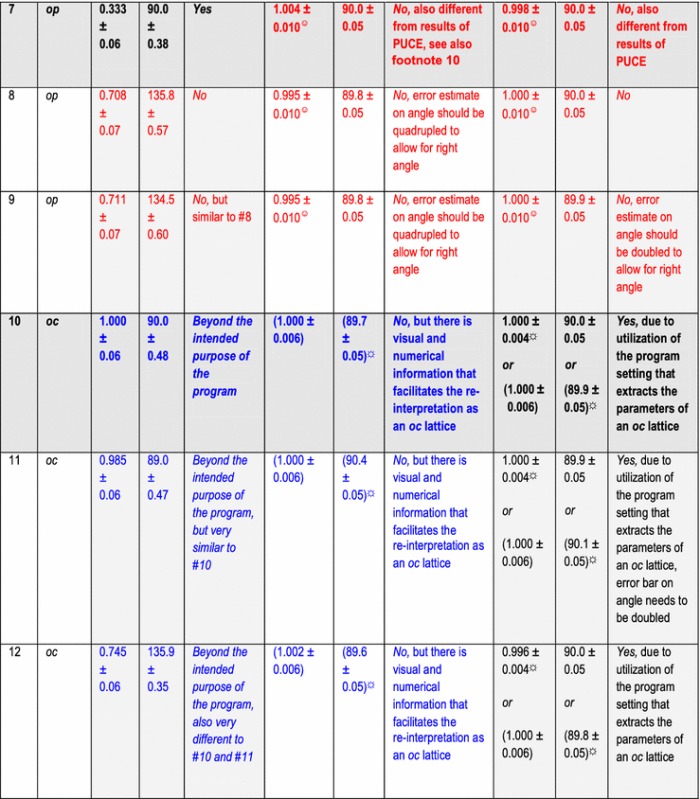
Summary of results obtained by the three programs in their *default* settings that were put to the task of extracting lattice parameters from images #1 to #12 in Fig. 2 (without any re-calculations/re-interpretations)

The capital case letters BL stands for the Bravais lattice. The two small case letter abbreviations of the Bravais lattice type in the second column follow the crystallographic standard convention [25], see also Fig. 1 and the “Bravais lattice types in two dimensions” section. The listed Bravais lattice types are those that the images possess per design on the basis of their 2D-periodic motifs and lattice parameters. The ^♦^ sign in the table headline refers to the primitive sublattice of a rectangular centered Bravais lattice. When there are parentheses around an entry in the columns of the unit cell angles and lattice vector magnitude ratios, the entry within them refers to a primitive sub-unit of a two times larger rectangular centered unit cell in direct space. Such entries exist only for the CrysTBox and CRISP programs since the PUCE program is not designed to give the user any feedback if the extracted unit cell of a more or less 2D-periodic image might be of the rectangular centered Bravais lattice type. The ^☼^ signs refer to translational pseudo-symmetries of the special kind or a metric specialization by design. The ^☺^ signs refer to artifacts of combinations of motif-based and translational pseudo-symmetries of the first kind

**Table 4:**
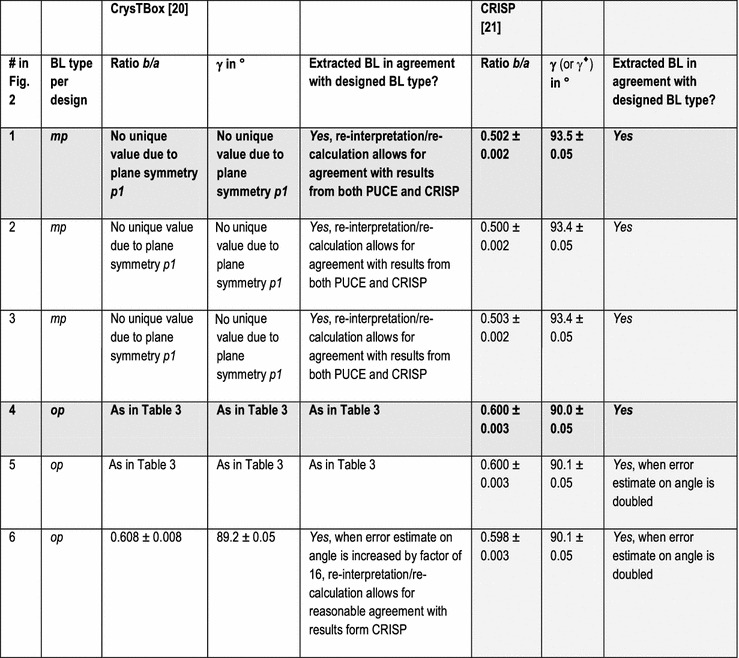

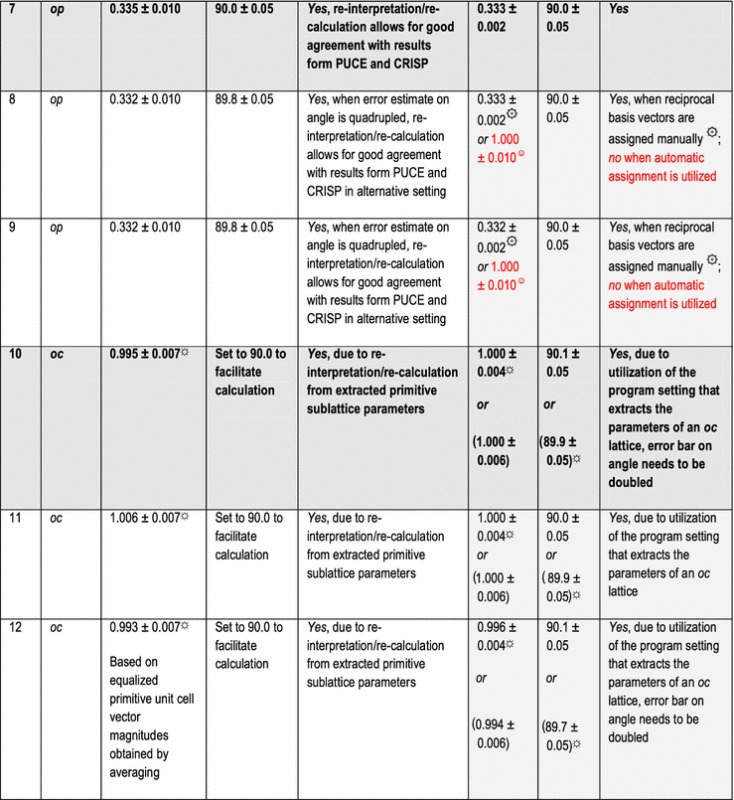
Summary of lattice parameter extraction results obtained with CrysTBox by re-calculation/re-interpretation on the basis of the images’ discrete Fourier transform amplitude maps and with CRISP in *non*-*default* settings

Entries in parentheses exist now only for the CRISP program, where just one extra click is required to test for the existence of a rectangular centered unit cell whenever the extracted lattice vector magnitudes are very close to each other. The ^۞^ signs refer to reciprocal lattice vector assignments by hand (rather than by the default automatic setting) in the CRISP program

**Table 5:**
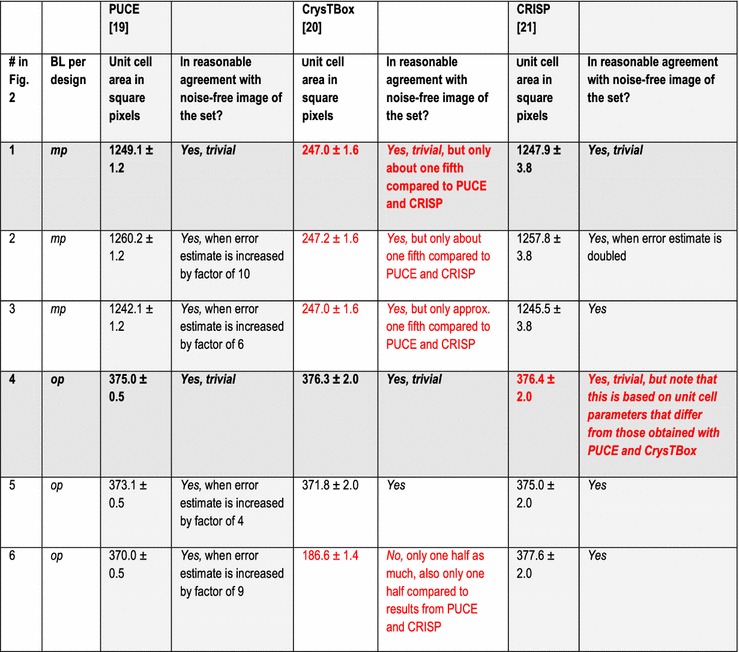

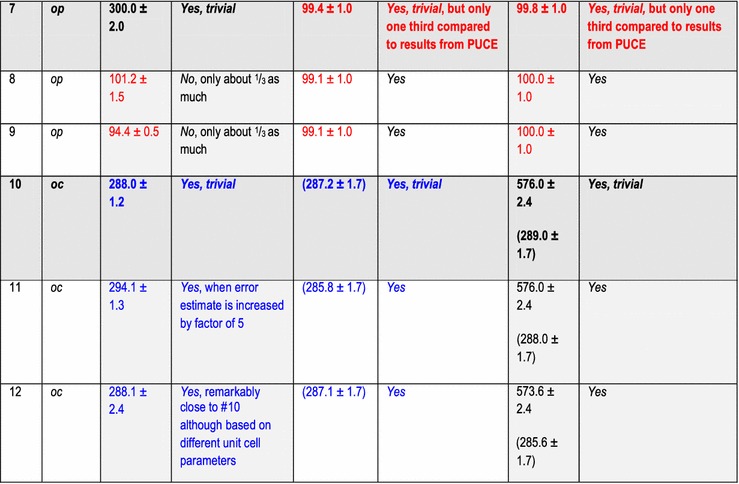
Continuation of the summary of results that were obtained by the three algorithms/programs in their *default* settings put to the task of extracting the lattice parameters of images #1 to #12 in Fig. 2 (without any re-calculations/re-interpretations)

Entries in parentheses refer to a primitive sub-unit cell of a two times larger rectangular centered unit cell in direct space

**Table 6:**
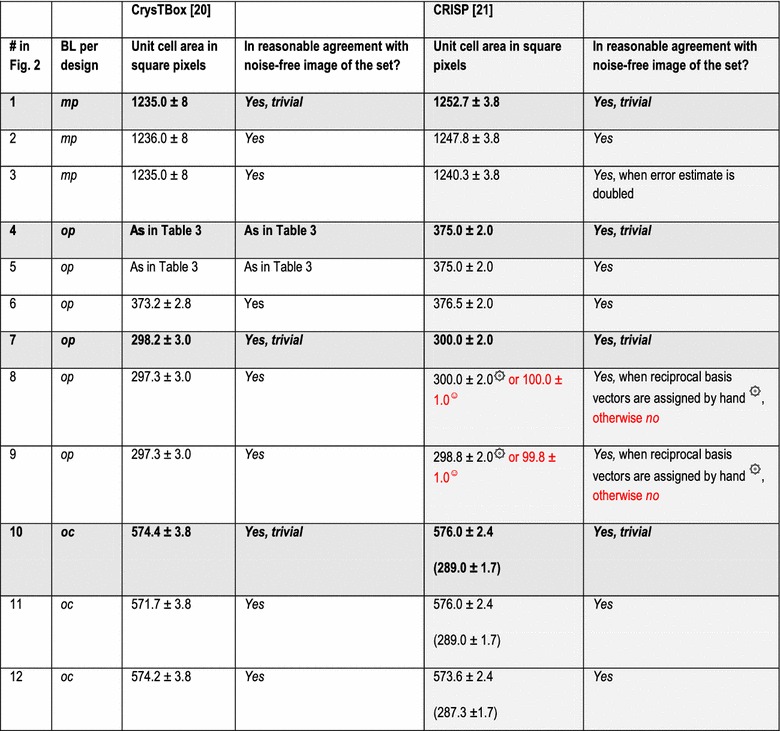
Continuation of the summary of results that were obtained with CrysTBox by re-calculation/re-interpretation on the basis of the images’ discrete Fourier transform amplitude maps and with CRISP in a *non*-*default* setting

Analogous to Table 4, entries in parentheses exist only for the CRISP program, where just one extra click is required to test for the existence of a rectangular centered unit cell whenever the extracted lattice vector magnitudes are very close to each other

While Tables 3, 4 list the extracted direct space lattice vector magnitude ratios and extracted lattice angles, Tables 5, 6 do the same for the derived direct space areas of the unit cells to which the lattice parameters correspond. The latter two tables are to be read as continuations of the former two tables. The first two columns of Tables 5, 6 are, therefore, identical to their counterparts in Tables 3, 4. The second column in all four of these tables lists the Bravais lattice types that the images of Fig. 2 possess per design in compliance with their 2D-periodic motifs and plane symmetry groups. Note that there are comment columns on major aspects of the listed results in all of the four tables.

The numerical outputs of the CRISP program were taken as significant numbers, i.e., the precision of each of the output parameters is assumed to be smaller or at most equal to 50% of the last digit of the numerical results. This corresponded in our review to a lattice vector magnitude extraction precision of one twentieth of one pixel, which seems reasonable at first sight for systematic error free synthetic data that underwent two least-squares refinement cycles in reciprocal space for the reciprocal lattice assignment in CRISP.

That level of presumed precision seems to be too high for the lattice angles, at least for the noisy images, as noted in a few places in Tables 3, 4 for both the CRISP and the CrysTBox programs. In verbal discussions of agreements or disagreements of results from the different programs for noisy images below, we consider, therefore, extracted lattice angles that vary by up to 0.5° as still in “reasonable” agreement with their counterparts that are imposed by the Bravais lattice types (as known per design).

Derived quantities such as the *b/a* ratios in Tables 3, 4 as well as the areas of the unit cells in Tables 5, 6 acquired precision measures by the standard propagation law of estimates, i.e., the sum of absolute values of the partial differentials at the extracted values times 50% of the last digit of the numerical result (significant number) that are associated with the extracted lattice parameters.

As a results of direct space lattice vector magnitude extraction precisions of 0.05 pixels for lattice vector magnitudes and 0.05° for lattice angles (for the algorithms behind the CRISP and CrysTBox programs), we obtain for image #5, for example, relative errors of slightly more than 0.5% for both the lattice vector magnitude ratios (Tables 3, 4) and unit cell areas (Tables 5, 6). This is consistent with the better than 1% (even down to approximately 0.1% [12]) accuracy reported for extracted reciprocal lattice vector magnitudes from experimental images in Ref. [20]. Our assumption that the CrysTBox program can deliver precisions for the extracted lattice parameters on the same order of magnitude as the CRISP program are also justified by these relative errors.

For the outputs of the PUCE program, a lattice vector coordinate uncertainty of 0.05 pixels seemed reasonable for noise-free images and we based our error propagation calculations on this assumed extraction precision. This is in reasonable agreement with the stated sub-pixel precision of lattice vector magnitudes [19] most of the time and resulted in error estimates on the derived entries for this program in Tables 3 and 5 that are up to one order of magnitude larger than for the lattice vector ratios and lattice angles that we obtained with CRISP and CrysTBox.

For the unit cell areas, on the other hand, all three programs provided error estimates on the same order of magnitude. We finally rounded the entries for both the CrysTBox and PUCE programs in Tables 3, 4, 5 and 6 to the same numbers of digits as the entries for the CRISP program.

We list in Tables 3, 4 the angle between the direct space lattice vectors, *γ*, in degrees. An additional superscript on the *γ* angle, i.e., ^♦^, and an entry between parentheses in both tables refers to the primitive sublattice of a possibly existing rectangular centered (*oc*) Bravais lattice, which would possess a unit cell that is twice as large in area (in direct space) as the primitive sub-unit cell.

The ^☼^ signs in Tables 3, 4 refer to translational pseudo-symmetries of the special kind or a metric specialization of the primitive sublattice of a rectangular centered Bravais lattice, which cannot be identified from the extracted lattice parameters alone but which we know must be present as there were no fourfold rotation symmetries in the amplitude maps of the dFTs of images #10 to #12. (As the CrysTBox and the CRISP program both display the amplitude map of the dFT of a more or less 2D-periodic image as outputs, this kind of translational pseudo-symmetry or metric specialization can only be identified by these two programs so that ^☼^ signs only show up in the corresponding entries for these two programs in Tables 3, 4).

The ^☺^ signs in Tables 3, 4 refer to combinations of genuine motif-based and translational pseudo-symmetries that are particularly pronounced in the noisiest image (#9) of the corresponding set of test images, see Fig. 2. As mentioned already in the “Overview I: synthetic test image sets” section, the whole set of these test images (#7 to #9) possesses per design a rectangular (primitive) Bravais lattice with a *b/a* lattice parameter ratio of one third, but the added noise “washes out” the intensity differences of the three dots in the translation periodic motif so that the lattice vectors appear, essentially, to be of an equal magnitude in Fig. 2, resulting in unit cells that are apparently (visually) of the square Bravais lattice type. (These three images possess motif-based and translational pseudo-symmetries of the first kind per design as already mentioned above in the caption of Fig. 2).

As it happens, the direct space dot intensity differences are too small, even for the noise-free image of this set (#7), to be interpreted correctly in a qualitative sense by the CRISP program in its default setting, see Table 3. This setting comprises both (i) the largest possible square image area selection and (ii) the automatic reciprocal lattice assignment mode. With the intensity differences of the designed dots further diminished by added noise in images #8 and #9, CRISP in its default setting leads within error bars to the extraction of a square Bravais lattice in these two cases as well, which is obviously incorrect. A byproduct of CRISP’s failures to extract the second linearly independent shortest lattice vectors in its default setting are unit cell areas that are only one third of the unit cell that the PUCE program obtained for image #7, see Table 5.

When the CRISP program is, however, used in alternative/non-default settings, i.e., (i) largest possible circular image area selection and (ii) manual reciprocal basis vector assignment options, qualitatively correct lattice parameters and unit cell areas are obtained within reasonable error bars for all three images of this set, see Table 4, so that the rectangular (primitive) Bravais lattice type can be assigned as being the most likely translation symmetry type.

The results of the manual (non-default) reciprocal basis vector assignment settings of the CRISP program are marked by ^۞^ signs in Table 4. The corresponding results of the automatic reciprocal lattice assignment setting of this program are also listed in Table 4 and carry ^☺^ signs to indicate artifacts of combinations of motif-based and translational pseudo-symmetries of the first kind.

The three comment columns in Tables 3, 4 provide answers to the question if the extracted/derived results are for each of the tested programs in reasonable agreement with the Bravais lattice type that we know the images possess due to their design. Note that this question concerns only the Bravais lattice type rather than the actual values of the lattice parameters. The answers to this question are, therefore, qualitative in their nature and to be considered (non-definitive) likelihood statements.

A ‘no’ in any of these three columns is a marker for a qualitative failure to extract correct lattice parameters by the corresponding algorithm/program. A ‘yes’ in either of these three columns is to be considered as a marker for a qualitatively correct translation symmetry type extraction, although the extracted lattice parameters may still be in error in a quantitative way. This kind of qualitative agreement is on occasions expanded to a ‘yes, but …’ when there are disagreements between the numerical results that were obtained from images of the same synthetic test image set with either the same program or the other two programs.

Obviously, the images of the same test set as shown in the individual columns in Fig. 2 should within reasonable error estimates yield the same lattice parameters and derived results because these images possess the same translation symmetry per design. In the case of the two noisy images of each test set, essentially the same lattice parameters as those of the noise-free image of these sets should have been extracted by each of the programs within not precisely known error estimates.

Most striking about the entries in Tables 3, 4 on the one hand, and Tables 5, 6 on the other hand, are the numerous differences in the individual entries. If the three tested algorithms were close to the above-mentioned unattainable *ideal* algorithm [16] and their default settings and internal parameters were optimally chosen by the programmers for the processing of our set of synthetic test images, one would naively expect that their extraction results are at least for the noise-free images in very good agreement. Obviously, this is not always the case. Results that are obviously incorrect (within any reasonable error bars) are marked with red ink in Tables 3 and 5. Blue markings in these two tables refer to entries that are not obviously incorrect but may appear to be so due to a program’s inability to extract the lattice parameters of a rectangular centered Bravais lattice.

For the entries of the PUCE program in Tables 3 and 5, there are two red markings. There are also three markings with blue ink in these two tables for the entries of this program. They refer to images #10 to #12, where a rectangular centered Bravais lattice type has been implemented by design, albeit with either a metric specialization (#10) or extreme cases of a translational pseudo-symmetry of the special kind (#11 and #12) as visually apparent by the somewhat “squarish appearance” of these three images to a human being in Fig. 2.

There is nothing in the outputs of the PUCE program for these three images that would hint at either the presence of a rectangular centered Bravais lattice or a metric specialization or extreme cases of translational pseudo-symmetry of the special kind. The program is simply not designed for these kinds of assessments as partly attested to by its full name “Primitive Unit Cell Extraction”. The entries in blue ink in Tables 3 and 5 for the PUCE program are, therefore, not to be counted as obviously incorrect, so that seven out of nine, i.e., approximately 77.8%, of the lattice parameter extractions with this program are to be considered as yielding results that are in qualitative agreement with the per design known Bravais lattice types of the images.

The differences between quantitative results that were extracted from noise-free images and their noisy counterparts within test image sets are for the PUCE program typically larger than that for their counterparts that were extracted with the two programs that operate in reciprocal space, i.e., CrysTBox and CRISP. In general, there is a tendency for the discrepancies of the extraction results between the three programs to be more pronounced for the noisy images of the test image sets.

It may not be incidental that the two entries in red ink for the PUCE program in Tables 3 and 5 refer to the images that feature combinations of motif-based and translational pseudo-symmetries of the first kind (per design) that are exacerbated by Gaussian noise (#8 and #9) which turns the genuinely exiting symmetries into pseudo-symmetries of the second kind. The areas of the extracted unit cells of these two images in Table 5 are only about one third of the area of the unit cell of the noise-free (and, therefore, less pseudo-symmetric) image in this set, i.e., #7.

For the image with the metric specialization, #10, the primitive sub-unit of a rectangular centered Bravais lattice has been extracted with the PUCE program. Within an “extended” error bar of up to 1°, the lattice parameter extraction result from image #11 for the PUCE program is also compatible with the designed primitive sub-unit of the rectangular centered Bravais lattice, see Table 3. For the noisiest image in the set, #12, on the other hand, neither reasonable nor extended error bars allow for an agreement between the quantitative lattice parameter extraction results from the PUCE program and the qualitative nature of the primitive sub-unit of the rectangular centered Bravais lattice that this image possesses per design. It is remarkable, however, that the extracted unit cell area of image #12 is in very good agreement with the unit cell area that was extracted by this program from image #10, see Table 5. (We will comment on this further below).

With seven red and three blue markings in Tables 3 and 5, the CrysTBox program does not seem to perform well at first sight when the quantitative outputs of the program are not re-interpreted/re-calculated on the basis of the amplitude maps of the dFTs of the images. Tables 4 and 6 paint a very different picture precisely because of re-interpretations and re-calculations.

The two neither red nor blue markings for CrysTBox in Tables 3 and 5 signify extracted lattice parameters in qualitative agreement with the a priori known Bravais lattice types for approximately 22.2% (i.e., 2 of 9) of the images. For the entries of the CRISP program in its default setting, there are four red (and no blue) markings in Tables 3 and 5, corresponding to extracted lattice parameters in qualitative agreement with the a priori known Bravais lattice type for approximately 66.7% (i.e., 8 out of 12) of the test images.

It is striking that the agreement between the entries in Tables 4 and 6, i.e., those that have been obtained with non-default settings for CRISP and by means of re-interpretation/re-calculations of a majority of the results from CrysTBox, is much better than that between their counterpart in Tables 3 and 5. As a matter of fact, not a single entry in the former two tables needed to be marked in either red or blue ink when the reciprocal basis vectors were selected manually! This indicates that the default settings (and internal parameters) of CRISP and CrysTBox are not optimal for the synthetic test images of this review.

Better lattice parameter extraction results can, for example, be obtained with CrysTBox when images edges and unit cell axes are not aligned parallel to each other^10^ as is the case per design in most of the images in Fig. 2. This is due to streaking parallel to the image edges in the dFT implementation that this program employs.

The default “maximal possible square” image area selection feature of the CRISP program also leads to streaking, while the non-default “maximal possible circular” image area selection feature of the CRISP program suppresses it quite effectively. The better lattice parameter extraction results in Tables 4 and 6 attest to the fact that it is generally beneficial to select the maximal possible circular area of an image for the calculation of the discrete Fourier transform in the CRISP program. On the other hand, the information in approximately 21.5% of a square image is excluded from image processing routines by this non-default setting of CRISP. An alternative way to suppress streaking in the discrete Fourier transform that both programs would probably benefit from is described in Ref. [45].

The consequences of streaking in the dFT are clearly revealed by the results from image #4 in Table 3, where the parameters of an alternative, but less symmetric, and therefore incorrect translation symmetry type have been extracted by CRISP in its default setting. The extracted unit cell parameters of this image refer to the oblique Bravais lattice type, while parameters that are compliant with a rectangular (primitive) Bravais lattice type should have been extracted instead.

The inspection of the direct space outputs of the CRISP program reveals that an alternative set of twofold rotation points (in plane symmetry group *p2*) has been selected by this program in its default setting as the unit cell origin for image #4. The extraction of a different set of lattice vectors is the direct consequence of this origin choice which was triggered by streaking in the dFT. Because *p2* is a subgroup of *p2mm* [25], which this image possesses per design, this alternative origin choice ensures that a qualitatively correct unit cell area is obtained within its error bar for this image, see Table 5, in spite of the lattice parameters being obviously wrong, see Table 3.

The inspection of the amplitude map of the dFT of this image revealed that the shortest reciprocal lattice vector has been ignored by CRISP in its default setting and that the second and third shortest lattice vectors were instead chosen as reciprocal basis. The magnitude of the third shortest reciprocal lattice vector corresponds to the reciprocal of the diagonal of the direct space unit cell that image #4 possesses by design. The area of the direct space unit cell that is obtained for this particular reciprocal lattice basis assignment must, for geometric reasons, match that of the rectangular unit cell area that this image features per design.

When CRISP is employed to image #4 in a non-default setting, i.e., when the maximal possible circular image area is selected for the extraction of the lattice parameters so that streaking in the dFT is suppressed, qualitatively correct results are obtained, see Tables 4 and 6.

Note that the above-mentioned result of the PUCE program on image #12 may fall into the same “category” as the result of the CRISP program in its default setting for image #4. There are, however, no program outputs that would allow us to test this hypothesis. It is, however, notable that the extracted and derived lattice parameters are again compatible with the oblique Bravais lattice type, Table 3, while being qualitatively incorrect. The extracted lattice angle of 135.9° is remarkably close to what one would expect if the diagonal of the designed unit cell was taken as one of the unit cell parameters in direct space, i.e., 135.0°. The area of the derived unit cell of image #12 is remarkably close to the one that has been derived with the PUCE program for image #10, i.e., the noise-free image of this set, see Table 5.

The comment columns of Tables 5, 6 are of particular importance for images that possess per design the oblique Bravais lattice type and plane symmetry group *p1*, i.e., images #1 to #3. This is because there is no crystallographic origin convention [25] for this particular combination of plane symmetry group and Bravais lattice type (as already mentioned in the “Bravais lattice types in two dimensions” section), so that there is arbitrariness in the selection of the lattice parameters in direct space. One can, therefore, not decide solely on the basis of the entries in Table 3 for these three images and for all three tested algorithms if the extracted lattice parameters are in agreement. Any extracted or derived lattice parameter set must, however, represent one lattice point so that the areas of the derived unit cells must be of the same size, within reasonable error estimates, if the extraction results are to be qualitatively correct.

Armed with this insight, we note that the unit cell areas for images #1 to #3 that were derived on the basis of the extraction results of the CrysTBox program are very different from those that were derived on the basis of the extraction results of both the CRISP and the PUCE programs, see Table 5. We conclude, therefore, that the lattice parameters that the CrysTBox program extracted for the images that possess an oblique Bravais lattice per design (#1 to #3) are all in need of a re-interpretation, although they are at least consistent within this set of test images. The inspection of the amplitude maps of the dFTs of images #1 to #3 and of the corresponding maps of the other four images (#6 to #9) for which entries in red ink exist for the CrysTBox program in Tables 3 and 5 revealed that the reciprocal lattice assignment was not based on the two shortest reciprocal lattice vectors in the amplitude maps.

In reciprocal (Fourier) space, a human operator would always assign [10]* and [01]* labels to the two shortest lattice vectors in the amplitude map of a dFT regardless of their intensity when she or he intents to extract the direct space lattice parameters for subsequent Bravais lattice type assignments. As already mentioned in the “Overview II: tested algorithms/computer programs” section, the CrysTBox program follows a different strategy. A reciprocal lattice is assigned by this program on the basis of it being highly precise rather than outlining one *genuine* reciprocal unit cell. This means that the two shortest reciprocal lattice vectors may not be selected as reciprocal basis when they have a rather low intensity.^11^

Since we saw from the amplitude map of the dFT of an image which reciprocal lattice spots had been selected as the reciprocal basis vectors by the CrysTBox program, we made re-interpretations and re-calculations of the corresponding direct space lattice parameters and the derived unit cell areas. The latter was particularly easy as we only needed to count the number of reciprocal lattice nodes that correspond to one CrysTBox determined reciprocal unit cell and multiply the derived direct space unit cell areas with the corresponding factor.

For images #1 to #3, there are four extra reciprocal lattice nodes that are completely included within the four lattice nodes that outline one reciprocal unit cell that CrysTBox has assigned. This means the four extra reciprocal lattice nodes count full because they are completely included within the algorithm assigned reciprocal unit cell. The four reciprocal lattice nodes that outline the assigned reciprocal unit cell itself count, on the other hand, just for one quarter of a full node each, because they are each shared with three other reciprocal unit cells. Four times one quarter plus 4 sums to 5 as the factor by which the direct space unit cell areas of these images as listed in Table 5, have been underestimated by the implemented CrysTBox assignment routines.

The corresponding entries for these three images as extracted by the CrysTBox program in Table 5 need, therefore to be multiplied by five in order to be listed in Table 6. In other words, the four nodes that outline the assigned reciprocal lattice unit cells in the amplitude maps of the dFT of these images account for a five times smaller unit cell in direct space.

Analogously for the other images for which there are red ink entries in Tables 3 and 5 for the CrysTBox program, the derived unit cell area for image #6 is to be doubled and for images #7 to #9 are to be tripled for listings in Table 6. The re-calculation of the direct space lattice parameters on the basis of the amplitude maps of the dFTs (as obtained with CrysTBox) of images #6 to #9 is also straightforward.

In the case of image #6, there is one extra reciprocal lattice node completely included within the reciprocal lattice cell that the CrysTBox algorithm assigned and this node is located parallel to the a* axis. The direct space lattice vector magnitude along the ***a*** axis is, therefore, to be doubled so that the listed *b*/*a* ratio for this image in Table 3 is to be cut in half for a listing of the corresponding entry in Table 4.

For images #7 to #9, there are two extra reciprocal lattice nodes completely included within the reciprocal lattice cells that the CrysTBox algorithmic implementation came up with and they are both located parallel to the a* axis. The entries for the *b*/*a* ratios of these three images in Table 3 are, therefore, to be reduced to one third each for listings of the corresponding entries in Table 4.

For images #1 to #3 there is per design no crystallographic convention for the origin of the unit cells (as mentioned above). There are, therefore, no unique sets of direct space lattice vectors so that there are consequently no unique *b*/*a* ratios for the entries for these three images for the CrysTBox program in Table 4.

Probably due to the motif-based pseudo-symmetry of the first kind that apparently “fixes” the origin to the positions of the pseudo-twofold rotation points in these three images, both the PUCE and the CRISP program extracted lattice parameters in good agreement with each other as listed in Table 3. This resulted also in good agreements between the direct space unit cell area listings for both programs in Table 5.

As anticipated above in the “Overview I: synthetic test image sets” section, a motif-based pseudo-symmetry of the first kind that apparently does not change the Bravais lattice type does not present a challenge to lattice parameter extraction programs. The combination of motif-based and translational pseudo-symmetries of the first kind in images #7 to #9, on the other hand, which apparently does change the Bravais lattice type resulted for both the CrysTBox and the CRISP programs in entries marked in red ink in Tables 3 and 5.

For image #8, a lattice of the rectangular (primitive) reciprocal Bravais lattice type was clearly visible in the amplitude map of the dFT of the image when processed with CRISP. This program would however in its (default) automatic reciprocal lattice assignment setting ignore the first two weak peaks in the amplitude map of the dFT of image #8 and extract parameters of a pseudo-square Bravais lattice instead regardless of whether or not the maximal circular or square area of the image was selected for the processing, see corresponding entries in Tables 3, 4.

When the reciprocal basis vector assignment was, on the other hand, made in the alternative manual setting, rectangular vectors of uneven magnitudes were handed over to the rest of the algorithms of the CRISP program so that qualitatively correct results were obtained, see Table 4. The consequences of the two different modes of reciprocal basis vector assignments of the CRISP program for the derived unit cell areas of image #8 are shown by the corresponding entries in Tables 5, 6. While the particulars of the entries in Tables 3 and 5 for this image are analogous to those that were derived with the CrysTBox program, it was the option in the CRISP program that allowed for manual reciprocal basis vector assignment that made all the difference to arrive at qualitative correct results as listed in Tables 4 and 6.

For the noisiest image of this test image set, image #9, the first two weak peaks in the dFT amplitude map of the CRISP program were almost indiscernible to us due to the very noisy background although one could still make them out if one “knew” that they must be present. The results for this image are, therefore, analogous to that of image #8 as we also utilized the alternative (non-default) manual reciprocal lattice assignment feature of the CRISP program in order to compile the entries for image #9 in Tables 4 and 6.

It is remarkable that the noise-free image of this test set, image #7, follows the same pattern in as far as the CRISP program in its default setting is concerned, see Tables 3 and 5. While a human being is visually capable to discern the underlying rectangular (primitive) Bravais lattice type that this image possesses per design, the combination of motif-based pseudo-symmetry and translational pseudo-symmetry of the first kind seems to be strong enough to “fool” both the CrysTBox and the CRISP program in their default settings. In the non-default setting of the CRISP program which involves the selection of the maximal circular area for further image processing, the automatic (default) reciprocal basis vector assignment function sufficed to arrive at qualitative correct results for image #7, see Tables 4 and 6.

The extraction results from the images that possess per design the rectangular centered Bravais lattice, i.e., #10 to #12, require a separate discussion as they possess either a metric specialization or extreme cases of translational pseudo-symmetry of the special kind. The “detached lattice symmetry” (see footnote 3) or very pronounced pseudo-symmetry (of the special kind) was easy to detect in the amplitude map of the dFT as provided by the CrysTBox and CRISP programs. For a thorough elucidation of different types of pseudo-symmetries, one needs to determine the most likely Laue classes and plane symmetries of the test image set in addition [18]. We will report on this elsewhere.

While the lattice parameters needed to be re-calculated from the outputs of the CrysTBox program for images #10 to #12, the CRISP program possesses (as already mentioned above) an alternative setting to test for the existence of a rectangular centered Bravais lattice type whenever the ratio of the magnitudes of the shortest reciprocal lattice vectors is approximately unity. We utilized this feature under both the default and non-default settings of the CRISP program to arrive at qualitatively correct results in both cases, see Tables 3, 4, 5 and 6.

We re-calculated the entries for the rectangular centered Bravais lattices for listings as entries for images #10 to #12 for the CrysTBox program in Tables 4 and 6 from the extracted primitive sublattice parameter sets as listed in Table 3. The sets of lattice parameters and derived unit cell areas of these three images were, after the re-interpretation, in good agreement with those that were obtained with the CRISP program by the more direct route.

We have to note in passing that the results of the PUCE program depended sensitively on image format conversion processes that were performed prior to the lattice parameter extractions. Some of the utilized image format conversion programs changed the nature of the noise inadvertently so that it was no longer Gaussian.

Surely, any image format conversion software should not do this kind of thing because it is equivalent to the inadvertent introduction of systematic errors into the synthetic test images. The results of both the CrysTBox and the CRISP program are, on the other hand, quite insensitive to image format conversions that were done to their inputs for all of the cases we studied in this review. This is probably due to the built-in “noise-filtering feature” of lattice parameter extraction algorithms that work in reciprocal space.

Note finally that the results from the moderately noisy image #5 are within reasonable error bars in qualitative agreement across all three programs/algorithms in their default settings, see Tables 3 and 5. A re-interpretation and re-calculation of the result of CrysTBox was not indicated by the amplitude map of the dFT of this image. The results of the CRISP program are actually identical in its default and non-default settings for image #5. The combination of these three features makes image #5 apparently the one from which it was easiest to extract qualitatively correct lattice parameters. The large number of unit cell repeats in this image probably played a role in this, in spite of the added noise. Based on the results of the CRISP program in its default setting for image #4 in Table 3, we must conclude that it was actually the noise-free image of the corresponding set that proved to be more challenging to the tested lattice parameter extraction algorithms in their default settings.

For image #5, one would be justified to average the lattice parameter extraction results and what has been derived from them over the three different algorithms and to obtain a higher accuracy and precision. Indeed the average *b*/*a* ratio for this image is 0.601 ± 0.011, the average lattice angle is 89.93° ± 0.08°, and the average unit cell area is 373.3 ± 1.0 square pixels as obtained from the combination of the results of the three programs in their default settings. For comparison, the design parameters for image #5 are: *b*/*a* ratio = 0.60, lattice angle = 90.0°, and unit cell area = 375 square pixels. The agreements between the averaged extraction results and the design parameters are for this particular image pretty good, but our initial error estimates that took the outputs of the CRISP program as significant numbers were, as Tables 3 and 5 clearly reveal, too optimistic.

As a matter of fact, the initial error estimates were throughout the whole review far too optimistic. Ten to twenty times larger error estimates than the initially assumed significant number outputs of the CRISP program are obviously more realistic given the totality of the results discussed in this section. Typically extracted lattice parameter magnitudes are, therefore, at least for the kinds of noisy images that are shown in Fig. 2 only accurate within approximately 2% and extracted lattice angles only accurate within approximately 1°.

## Summary and conclusions

Three different algorithms (as implemented in three different computer programs) were put to the task of extracting lattice parameters from four sets of synthetic test images that were 2D periodic per design but also contained images that were noisy so that all site and translation symmetries were broken. While one of the images in each of these sets was free of noise (and also free of systematic errors so that it was perfectly 2D periodic), independent Gaussian noise of mean zero and a standard deviation of 10 or 50% of the maximal pixel intensity was added to the individual pixels of that image in order to create two noisy images for each set of test images. While the added noise obscures the translation and site symmetries in these images, it obviously cannot change them in a systematic way. The presence of noise is supposed to present a greater challenge for any computer program to extract accurate lattice parameters with a high precision.

Our sets of calculated test images can be considered to be equivalent to images that were detected with different signal-to-noise ratios by an instrument that is free of systematic imaging errors. The signal in the images of one test set is then a combination of individual pixel intensities that obey designed restrictions that are set by the combination of the chosen plane symmetry group, i.e., a combination of translation and site symmetries, with the metric properties of the unit cell.

A (non-existing) ideal algorithm for the extraction of lattice parameter information applied to any one of these test images would have quantified the magnitudes of the two basis lattice vectors and the angle between these vectors accurately to the values that were put into the images’ designs. The translation symmetry/Bravais lattice type that is to be inferred from these parameters would also be the one that was put into the images’ designs.

Moreover, an ideal algorithm would not have introduced systematic errors into the geometric extraction results and delivered the same results for the three individual images of each test set. An increase of the error estimates on the lattice parameters would be expected with an increasing amount of noise in the images as the ideal algorithm would propagate random errors faithfully. This kind of an ideal algorithm could be considered as the (non-existing) *definitive* algorithm for the extraction of geometric-structural features from noisy images for the task at hand.

A minor complication arose in our review by the fact that none of the three tested programs provides explicit statements on error bars. We were, therefore, initially forced to take the numerical outputs of the computer program that provides the fewest number of digits for the extracted lattice parameters as significant numbers. This was the CRISP program, which had also the best overall test performance. The resulting error estimates were then also used for the outputs of the other program that extract lattice parameters in reciprocal space (i.e., CrysTBox). The error bars on the unit cell areas and other derived results were for these two programs obtained by standard error propagation calculations. As a result of this review, we have to conclude that our initial error estimates were way too optimistic by a factor of ten to twenty for the noisy images at least.

For the program that allows for lattice parameter extractions in direct space (i.e., PUCE), we made a reasonable assumption for the precision with which the Cartesian coordinates of the start and end points of lattice vectors could be extracted from the images. For quantities that were derived from the extracted coordinates, e.g., the ratio of the magnitudes of the lattice vectors, the lattice angle, and the area of the unit cell, the error estimates of the coordinates were propagated to the derived end results.

Contrary to our expectations, the sets of lattice parameters that the three programs extracted in their default settings from the same image disagreed in the vast majority of cases within both the originally anticipated and more reasonable error estimates. On the one hand, this fact reflects positively on the nature of the test images in the sense that they present tough challenges to lattice parameter extraction algorithms because many of them possess pseudo-symmetries of the first or special kind per design. This fact, however, also reflects somewhat negatively on the tested computer programs/algorithms because most researchers (including the programmers) would probably expect them to perform much better for the tasks at hand.

The main thrust of this paper was, however, not at all a ranking of the relative performance of the three tested programs. The test performances of the corresponding three different algorithm implementations are supposed to serve collectively as an illustration of the fact that there is simply no *definitive* extraction algorithm for geometric-structural features in all real-world applications. Nevertheless, a very brief summary of the test performances of the three computer programs is in order in this final section of this review.

Of the three tested programs, the one that has been around for more than a quarter of a century as a windows executable, i.e., CRISP, performed best. The CRISP program is also the only one of the three tested programs that allows for a direct route to the extraction of the parameters of rectangular centered Bravais lattices. Plane symmetries and Laue classes of more or less 2D-periodic images can also be determined with this program (in a somewhat subjective manner) so that it is the only one of the three tested programs that offers a systematic route towards elucidating pseudo-symmetries of the first and special kind.

The application of the CRISP program in non-default settings resulted in extracted lattice parameters that were entirely consistent with the designs for all test images. This could be due to both its classical 2D-crystallography approach^12^ and the noise-filtering function of the Fourier transform.

Naturally, lattice parameter extraction from noisy images is more difficult in direct space so that the PUCE program is at a disadvantage in this task in comparison to the other two programs. For three of the four noise-free images, the PUCE program extracted lattice parameters in very good agreement with the results from the CRISP program and the a priori known Bravais lattice types. In the fourth case, where there was a rectangular centered Bravais lattice type per design, the PUCE program extracted the parameters of its primitive sublattice (because it is designed to extract primitive lattice parameters only).

The lattice parameters that the CrysTBox program extracted in its default setting had to be re-interpreted/re-calculated in 10 out of 12 cases. The reason for this was in seven cases the implemented strategy of this program to assign a highly precise reciprocal lattice in reciprocal space that does not need to outline the smallest reciprocal lattice unit cell. This meant ignoring the shortest reciprocal lattice vectors when they had small amplitudes in the corresponding map of the dFTs of these images. After re-interpretation/re-calculation, all results from the CrysTBox were qualitatively correct. Also the CrysTBox program is optimized for work with electron diffraction patterns rather than more or less 2D-periodic images. This program possesses, in addition, many non-default settings that we did not test as part of this review.

For the detection of pseudo-symmetries of the first and special kind, the user benefits greatly when a computer program displays the amplitude map of the discrete Fourier transform of a more or less 2D-periodic image. This is because the dFT amplitude map displays the Laue class that corresponds to the underlying plane symmetry group. When there is no pseudo-symmetry of the first or special kind (and there is no metric specialization), there is also no obvious mismatch between (or detachment of) the visible point symmetry around the center of this map and the translation symmetry that is governed by the metrical properties of the extracted lattice parameters. While the CRISP and CrysTBox programs both possess such a feature, it is absent in the PUCE program as the latter works in direct space exclusively.

For dealing with pseudo-symmetries of all kinds, it is also helpful when a computer program allows the user to overwrite an automatic (default) assignment of the reciprocal basis vectors in the amplitude map of a dFT of a more or less 2D-periodic image. The CRISP program possesses this feature as well. The only negative thing that could be said about this program is that, typically, error bars on the extracted unit cell angles are at least one order of magnitude larger than one would expect based on the assumption that the CRISP program output are significant numbers. (The other two programs provide even more non-significant numbers as outputs).

We conclude finally that our testing of the lattice parameter extraction capabilities of three different algorithms (as implemented in three different computer programs) was useful because not all readers might have been aware that there are simply *no definitive* algorithms/computer programs for the extraction of geometric-structural features from *detected* images. As there is much more hierarchical geometric-structural information beyond Bravais lattice types that could be extracted from noisy images (and utilized, for example, for automated crystal phase and grain boundary symmetry type classifications), we hope to have brought the implications of Kanatani’s *no definitive geometric feature extraction algorithm/results in all real-world applications* dictum and his comments on the vast majority of computerized attempts to extract symmetries and other hierarchical geometric features from noisy images to the attention of the wider scientific community. This will hopefully lead to a more thoughtful *“use of established procedures in widely distributed software”* and a disengagement from *“the natural tendency of most people to prefer results in agreement with preconceived ideas”* as encouraged by the Committee on Statistical Descriptors of the International Union of Crystallography.

There are many more geometric-structural feature extraction programs to be written and thoroughly benchmarked with respect to each other on sophisticated test image sets in order to make progress collectively as a community towards the shared goals of model-based imaging, materials informatics, and the knowledge-based designs of new materials. Kanatani’s geometric AICs could become very useful in the pursuit of these overarching goals because they provide model parameter spaces of comparatively small dimensionalities, noise-level dependent rankings that are free of arbitrarily set thresholds, and are applicable whenever very small systematic imaging errors are negligible with respect to small random imaging errors with an approximately Gaussian distribution. Note that in the case of nested models, the noise level of an image does not even need to be estimated when one wants to find out which crystallographic model minimizes the unavoidable information loss (that is associated with the model’s usage as representation of the image data).

We also hope to have achieved the secondary goal of this paper so that misconceptions surrounding Bravais lattices in 2D and plane symmetry groups that existed in the wider scientific community are now cleared up. Addressing these shall hopefully foster the widespread application of G-AICs by microscopists, computer scientists, and applied crystallographers in the future.

## Abbreviations

1D: one dimension
2D: two dimensions
3D: three dimensions
AIC: Akaike information criterion
AICs: Akaike information criteria
dFT: discrete Fourier transform
dFTs: discrete Fourier transforms
FC: Fourier coefficient
FCs: Fourier coefficients
G-AIC: geometric Akaike information criterion
G-AICs: geometric Akaike information criteria
HRTEM: high-resolution transmission electron microscopy and microscope
PUCE: Primitive Unit Cell Extraction
SPM: scanning probe microscopy and microscope
STEM: scanning transmission electron microscopy and microscope
STM: scanning tunneling microscopy and microscope
TEM: transmission electron microscopy and microscope

## Footnotes

1 Pierre Curie’s well-known symmetry principle needs to be considered when the imaging process involves different kinds of physical effects and properties of crystalline materials.

2 Pseudo-symmetry refers in general terms to *“a spatial arrangement that feigns a symmetry without fulfilling it”* (M. Ruck as quoted and discussed in Ref. [18]). Pseudo-symmetries of the *first* kind (also referred to as genuine pseudo-symmetries) exist in addition to genuine symmetries that combine to the crystallographic group structure of a noise-free and perfectly 2D periodic image. This definition is in compliance with the one mandated for pseudo-symmetry by the International Union of Crystallography (IUCr) in its on-line dictionary, listed as Ref. [23]. Noise in an image results unavoidably in the turning of all genuine symmetries into pseudo-symmetries of the *second* kind. This includes the loss of the strict translation periodicity in both dimensions. When a translational pseudo-symmetry arises from the adding of noise to a perfectly 2D periodic (noise-free) image that features a metric specialization [24], we speak of a “special” kind of pseudo-symmetry. This is because that pseudo-symmetry is per our definition neither of the first nor of the second kind because a metric specialization is neither a genuine translation symmetry that combines with genuine point symmetries to a genuine crystallographically consistent symmetry group nor a pseudo-symmetry as defined by the IUCr. As a function of the signal-to-noise level of an image, it may become difficult or essentially impossible to distinguish between pseudo-symmetries of the first, special, and second kind for practical purposes. There remains, however, the clear distinction between the first and second kind of pseudo-symmetries per our definition or theoretical purposes (independent of the signal-to-noise level of an image). Translational pseudo-symmetry of the first kind manifests itself in reciprocal space in the form of very weak Fourier coefficient amplitudes of at least one of the shortest reciprocal basis vectors that may be barely recognizable in the presence of noise. Motif-based pseudo-symmetry of the first kind manifests itself in reciprocal space in the form of apparent point symmetries between certain Fourier coefficients amplitudes (and phase angles) that seem to exist in addition to those of the Laue class (and plane symmetry group) of a hypothetical noise-free version of an image. Pseudo-symmetry of the special kind manifests itself in a noisy image as a mismatch of the apparent translation symmetry and the Laue class (and plane symmetry) of a hypothetical noise-free version of an image. The mismatch is due to the “detachment” of the apparent metric lattice symmetry from the apparent site symmetries of the motif that it enables in both direct and reciprocal space.

3 The 2005 edition of Volume A of the International Tables for Crystallography considers metric specialization to represent a case of *“metrical pseudo-symmetry”* that may exist within experimental errors in some lower symmetric crystal *“accidentally”* at some temperature (and pressure) and would turn into a translational pseudo-symmetry of the first kind by means of anisotropic thermal expansion (or an anisotropic response to a pressure change) at some other temperature (or pressure) without a phase transition. At the exact temperature and pressure point of a metrical specialization, the lattice of a crystal is theorized to be *“detached”* from its crystal structure and space group. The words in italic font and quotation marks in this footnote are direct quotes from the above-mentioned most definitive reference text and websites in the field.

4 Site symmetries are the symmetries at individual points in the translation periodic motif. The translation periodic motif is the whole content of a unit cell and possesses either a symmorphic or a non-symmorphic symmetry. Symmorphic symmetry refers to the existence of at least one point in each unit cell that possesses the point group of the corresponding plane symmetry group as its site symmetry. The plane symmetry groups *pg*, *p2mg*, *p2gg*, and *p4gm* are, therefore, non-symmorphic.

5 Lattice points are a mathematical abstraction of both individual atoms and groups of atoms that form by themselves the crystallographic basis. Lattice points possess in 3D the symmetry of a sphere at rest, i.e. point symmetry ∞∞m, and represent the nodes of a mathematically abstract Bravais lattice. As individual atoms may possess the same point symmetry as lattice points when they are at rest (which is of course utterly unphysical), they may physically occupy positions with the same translation symmetry as the nodes of a Bravais lattice within a “one element crystal” structure. The crystallographic basis is then just a single atom, which is typically of a metallic element. Groups of atoms, on the other hand, possess lower point symmetries than ∞∞m so that one cannot imagine that they might be physically located at positions that correspond to the nodes of abstract Bravais lattices. The crystallographic basis for the crystal structure is then the whole group of atoms. In 2D crystallography, this group of atoms is also called the translation periodic motif. The widespread confusion between lattice points and individual atoms stems from the fact that both entities may possess the same point symmetry (as a time average only in the case of single atoms) so that they can be located at positions with the same combination of translation and site symmetries. As symmetry is involved, i.e. an abstract mathematical concept, the confusion is in 3D analogous to that between a space group type as a mathematical abstract concept and a crystal structure that possesses only as time average the individual symmetries of the same space group type.

6 All Laue classes in 2D contain twofold rotation points just as all Laue classes in 3D contain inversion centers. The point symmetries of the amplitude maps of discrete Fourier transforms display Laue classes of more or less 2D periodic images in reciprocal space.

7 This could also be the reason for inconsistencies in the extraction of frieze symmetries in gait sequences (recorded time series) of both a walking human avatar and a human being in Ref. [32] as discussed in Ref. [18].

8 Approximate site symmetries were extracted in direct space first for objects that were appealing to a human being in Ref. [40] and later on combined with Bravais lattice types for classifications of more or less 2D periodic images into plane symmetry groups. While this is a viable route that includes the fixing of the unit cell origin as a byproduct, it will probably be difficult to automate. Complications due to noise, defects, translational and motif-based pseudo-symmetries of the first kind, as well as a metric specialization of the translation symmetry were not addressed in a systematic way in Ref. [40].

9 Default settings and internal parameters (such as arbitrarily set thresholds) of programs have been implemented by programmers because they have empirically been found to work sufficiently well for the majority of images from which geometric-structural features are to be extracted. The criterion for a useful default setting and internal parameter is that the extraction results are to be obtained with a minimum of user interactions (e.g. clicks with the mouse). The pre-setting of defaults and internal parameters saves the novice user’s time when a computer program is employed on images that are deemed to be typical. The non-existing definitive algorithm would be able to deliver unbiased geometric-structural feature extraction results for any image that belongs to the class for which the algorithm has been designed.

10 Doctor Miloslav Klinger of the Institute of Physics of the Czech Academy of Sciences was so kind as to run a rotated version of this image through the new β-version of his CrysTBox program and shared his results with us. A rotation around the image center by approximately 10° with respect to the fixed image edges resulted in the extraction of a pseudo-square lattice with relative precisions of the lattice vector magnitudes of better than 0.2%. The motivation for this rotation was to break the parallel alignment of the image edges and the unit cell edges of the original image design.

11 This can be a very useful feature when there is more than one reciprocal lattice in the amplitude map of the discrete Fourier transform of an image. This kind of a situation would arise when an image contains information from more than one crystal. If one of two crystals occupied a much larger area in the image than the other crystal, e.g. there was some small crystalline inclusion in a crystalline matrix, there would indeed be two sets of Fourier coefficients with significantly different intensities in the amplitude map of the discrete Fourier transform of that image.

12 Note that this approach resulted in the award of the 1982 Chemistry Nobel prize to Sir Aaron Klug *"for his development of crystallographic electron microscopy and his structural elucidation of biologically important nucleic acid–protein complexes".*
